# Inhibition of nuclear factor (erythroid-derived 2)-like 2 promotes hepatic progenitor cell activation and differentiation

**DOI:** 10.1038/s41536-021-00137-z

**Published:** 2021-05-26

**Authors:** Francesco Bellanti, Giorgia di Bello, Giuseppina Iannelli, Giuseppe Pannone, Maria Carmela Pedicillo, Luke Boulter, Wei-Yu Lu, Rosanna Tamborra, Rosanna Villani, Gianluigi Vendemiale, Stuart J. Forbes, Gaetano Serviddio

**Affiliations:** 1 Centre for Experimental and Regenerative Medicine, Department of Medical and Surgical Sciences, University of Foggia, Foggia, Italy; 2 Anatomical Pathology Unit, Department of Clinical and Experimental Medicine, University of Foggia, Foggia, Italy; 3 MRC Human Genetics Unit, Institute of Genetics and Molecular Medicine, University of Edinburgh, Edinburgh, UK; 4 Centre for Liver and Gastrointestinal Research, Institute of Immunology and Immunotherapy, University of Birmingham, Edgbaston Birmingham, UK; 5 MRC Centre for Regenerative Medicine, University of Edinburgh, Edinburgh, UK

**Keywords:** Stem cells, Cell biology

## Abstract

The stem cell ability to self-renew and lead regeneration relies on the balance of complex signals in their microenvironment. The identification of modulators of hepatic progenitor cell (HPC) activation is determinant for liver regeneration and may improve cell transplantation for end-stage liver disease. This investigation used different models to point out the Nuclear factor (erythroid-derived 2)-like 2 (NRF2) as a key regulator of the HPC fate. We initially proved that in vivo models of biliary epithelial cells (BECs)/HPC activation show hepatic oxidative stress, which activates primary BECs/HPCs in vitro. NRF2 downregulation and silencing were associated with morphological, phenotypic, and functional modifications distinctive of differentiated cells. Furthermore, NRF2 activation in the biliary tract repressed the ductular reaction in injured liver. To definitely assess the importance of NRF2 in HPC biology, we applied a xenograft model by inhibiting NRF2 in the human derived HepaRG cell line and transplanting into SCID/beige mice administered with anti-Fas antibody to induce hepatocellular apoptosis; this resulted in effective human hepatocyte repopulation with reduced liver injury. To conclude, NRF2 inhibition leads to the activation and differentiation of liver progenitors. This redox-dependent transcription factor represents a potential target to regulate the commitment of undifferentiated hepatic progenitors into specific lineages.

## Introduction

The ability of stem cells to self-renew, maintain pluripotency, and lead tissue regeneration relies on the balance of complex signals in their microenvironment. The liver is characterized by unique regenerative capacity after consistent injury of various origin (genetic, toxic, metabolic, viral, or immunologic). Liver renewal during normal homeostasis or after an acute injury is driven by hepatocytes; however, in case of severe or protracted damage, liver regeneration is mediated by hepatic progenitor cells (HPCs), capable of differentiating toward both the biliary and the hepatocyte lineages^1–6^. HPCs are placed in niches located within the smallest branches of the biliary tree, at the interface between the hepatic parenchyma and the portal tract, named Canals of Hering^7^. In a quiescent state, the niche microenvironment keeps the progenitor phenotype and inhibits cell differentiation. Several types of liver damage trigger the “ductular reaction”, in which specific alterations of the niche microenvironment promote the differentiation of HPCs toward a hepatocyte or cholangiocyte phenotype^4^. HPC activation is the first stage in progenitor-dependent regeneration, thus a comprehensive knowledge of the mechanisms by which HPCs start to proliferate and differentiate may be determinant to develop new therapies for liver disease.

The persistent damage characteristic of several liver diseases leads to a disruption of redox balance caused by overproduction of reactive species^8^. Oxidative stress induces hepatocyte senescence with consequent cell cycle arrest and impaired liver regeneration^9^. Reactive species modulate and are modulated by several factors including the nuclear factor (erythroid-derived 2)-like 2 (NRF2), Forkhead box O (FoxO) family, glycogen synthase kinase (GSK-3β), the PR domain containing 16 (PRDM16), peroxisome proliferator-activated receptor gamma coactivator 1-alpha (PGC1α), the p53 (TRP53) tumor suppressor, Wnt and nucleoredoxin (Nrx)^10–16^. These transcription factors may be involved in the regulation of quiescence/self-renewal/differentiation of several stem cell lines^17^. However, to date a direct regulation of HPCs by redox signaling has not been demonstrated.

This study analyzed the oxidative damage in models of liver injury characterized by biliary epithelial cells (BECs)/HPCs activation and defined the impact of redox balance perturbation on HPC fate. We then identified NRF2 as the main transcription factor involved in the activation of HPCs. To confirm the key role of this redox-dependent factor and to investigate its therapeutic potential, we explored the effects of NRF2 modulation on a xenograft transplantation model using human cells with features of HPCs.

## Results

### Hepatic oxidative stress is associated with BEC/HPC activation

**Fig. 1:**
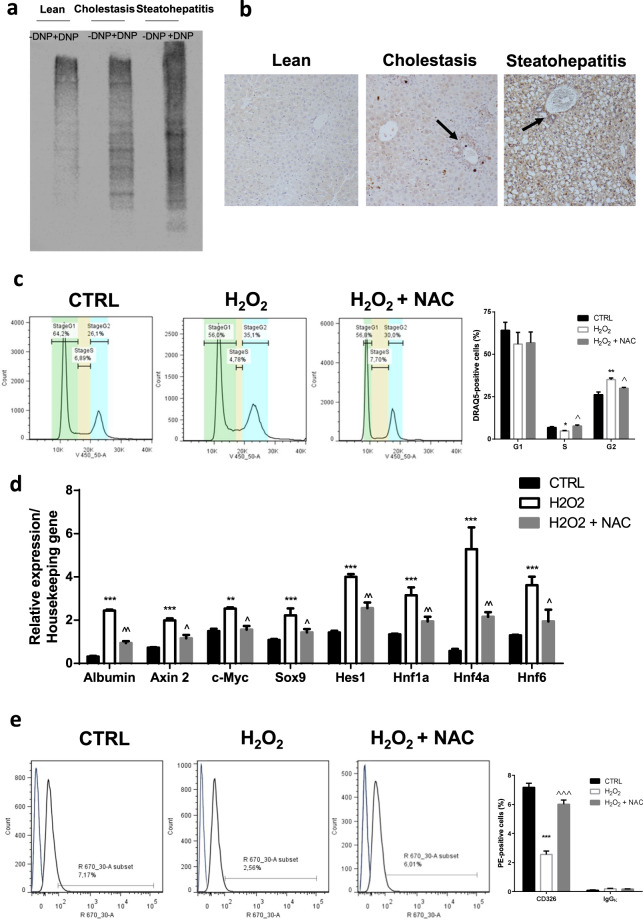
HPCs are activated by oxidative stress. **a** Representative oxyblot analysis of liver homogenates from mice fed a control chow (lean), 3,5-diethoxycarbonyl-1,4-dihydrocollidine diet (cholestasis), or methionine-choline-deficient diet (steatohepatitis); *DNP* dinitrophenylhydrazine. **b** Representative images showing immunohistochemical detection of 4-hydroxy-2-nonenal (HNE) in the liver of mice fed a control chow (lean), 3,5-diethoxycarbonyl-1,4-dihydrocollidine diet (cholestasis), or methionine-choline deficient diet (steatohepatitis) (magnification ×200). Black arrows indicate the portal tracts, where the canals of Hering are located. **c** Cell cycle analysis in BECs/HPCs exposed to 1 μM H_2_O_2_ or 1 μM H_2_O_2_ + 1 mM *N*-acetylcysteine (NAC) for 24 h. Data in the graph are represented as mean ± SEM of three independent experiments. Statistical differences were assessed by one-way ANOVA and Tukey–Kramer as post hoc test. *p < 0.05 vs CTRL; **p < 0.01 vs CTRL; ^*p* < 0.05 vs H_2_O_2_. **d** mRNA expression of Notch and Wnt pathway targets in BECs/HPCs exposed to 1 μM H_2_O_2_ or 1 μM H_2_O_2_ + 1 mM *N*-acetylcysteine (NAC) for 24 h. Data in the graph are represented as mean ± SD of three independent experiments. Statistical differences were assessed by one-way ANOVA and Tukey–Kramer as post hoc test. **p < 0.01 vs CTRL; ***p < 0.001 vs CTRL; ^*p* < 0.05 vs H_2_O_2_; ^^*p* < 0.01 vs H_2_O_2_. **e** Expression of EpCAM in BECs/HPCs exposed to 1 μM H_2_O_2_ or 1 μM H_2_O_2_ + 1 mM *N*-acetylcysteine (NAC) for 24 h. The blue line represents rat IgG isotype control antibody. Data in the graph are represented as mean ± SEM of three independent experiments. Statistical differences were assessed by one-way ANOVA and Tukey–Kramer as post hoc test. ***p < 0.001 vs CTRL; ^^^*p* < 0.001 vs H_2_O_2_.

We initially evidenced the oxidative injury in two well-validated mouse models of BEC/HPC-derived biliary and hepatocellular regeneration^4,18^. The hepatic total carbonyl content, which evaluates the protein damage associated to reactive species, was higher both in the model of cholestasis and in that of steatohepatitis (Fig. 1a). We then immunohistochemically detected protein adducts with 4-Hydroxy-2-nonenal (HNE), a major component of lipid peroxidation, which were greater in the liver of both cholestasis and steatohepatitis models; of note, the HNE-protein adducts were mainly detected in the portal tracts (where the canals of Hering are located), and this was particularly evident in the model of cholestasis (Fig. 1b).

To investigate the effects of redox manipulation on self-renewal and activation, primary murine BECs/HPCs were grown in the presence of redox-active compounds. To increase the oxidative level, BECs/HPCs were exposed to reactive species with different action: H_2_O_2_ (simplest peroxide, strong oxidizer, second messenger), tert-butyl hydroperoxide (t-BuOOH, organic peroxide, strong oxidizer), buthionine sulfoximine (BSO, inhibitor of glutathione synthesis), and antimycin A (inhibitor of mitochondrial complex III). To render cells more reduced, the medium was supplemented with N-acetylcysteine (NAC, a strong antioxidant and a cysteine pro-drug that enhances the synthesis of glutathione). Acute toxicity of each substance was studied (Supplementary Fig. 1a), and the highest non-toxic dosage was used in subsequent experiments. Of interest, 5 days treatment of BECs/HPCs with all the pro-oxidative compounds was associated with a modified phenotype (polygonal shape, containing distinctly round nuclei with prominent nucleoli, and intercellular structures resembling hepatic canaliculi), reduced cell adhesion, and increased spreading (Supplementary Fig. 1b–d). The subsequent experiments were conducted by exposing BECs/HPCs to different concentrations of H_2_O_2_. This exposure revealed that sublethal amounts of H_2_O_2_ induced the activation of cell cycle and promoted replication. Indeed, BECs/HPCs treated with H_2_O_2_ exhibited higher percentage of G2 phase as compared with control; the addition of NAC resulted in unaltered G2 phase (Fig. 1c). Furthermore, H_2_O_2_ exposure induced the expression of target genes of both Wnt and Notch pathways, which are involved in biliary and hepatocyte regeneration, respectively^4^; this effect was strongly limited by NAC co-exposure (Fig. 1d). Finally, BECs/HPCs exposed to H_2_O_2_ lost the expression of the epithelial cell adhesion molecule (EpCAM), a transmembrane glycoprotein that is gradually downregulated along with maturation into hepatocytes^19^; this effect was not observed in HPCs co-treated with NAC (Fig. 1e, Supplementary Fig. 2a). The reduced expression of EpCAM observed in H_2_O_2_-treated ductular/HPCs isolated from models of ductular/HPC activation was not associated with the induction of apoptosis (Supplementary Fig. 2b, c). Taken together, these results suggest that the perturbation of redox balance by induction of a pro-oxidative environment may activate BECs/HPCs.

### NRF2 is the main redox-dependent transcription factor driving HPC fate

**Fig. 2:**
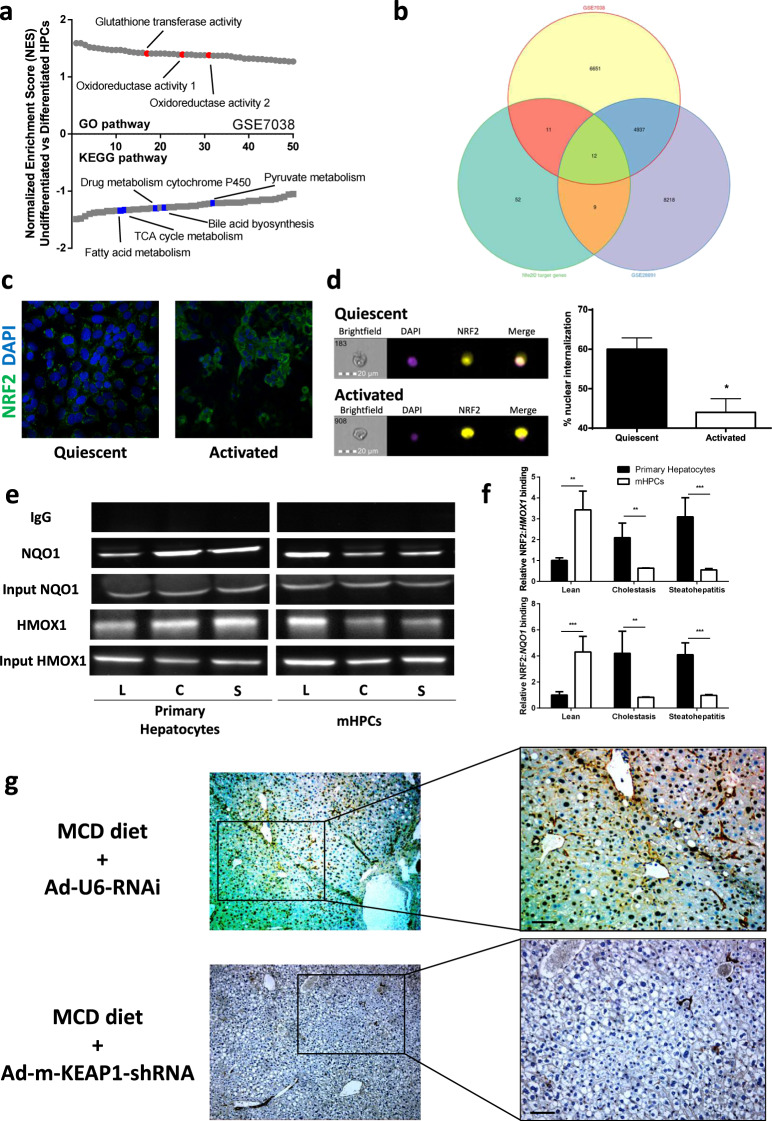
Nrf2 is determinant for HPC activation. **a** GSEA demonstrates up- and downregulated signaling pathways in undifferentiated HPCs as compared with differentiated cells. This panel shows the results of our analysis of microarray data from the publicly available GEO data set (accession number GSE7038), using Gene Ontology (GO) or Kyoto encyclopedia of genes and genomes (KEGG) enrichment. Signaling pathways were ranked based on normalized enrichment scores (NESs); positive and negative NESs indicate down- or upregulation, respectively, in undifferentiated HPCs. Specific pathways related to metabolic or redox pathways are highlighted in red and blue. **b** Area proportional venn diagram representing 84 common genes between the downregulated genes in transcriptomes originating from mature hepatocytes [GSE7038 and GSE28891] and NRF2 target genes (NCBI Entrez Gene Database, 4780). **c** Confocal microscopy was used to examine the nuclear localization of NRF2. Representative confocal images demonstrating nuclear (DAPI, blue) colocalization of NRF2 (green) in quiescent and activated BECs/HPCs; Z-stacks (1 micron) were taken and rotated in two dimensions. Differentiated cells clearly show no colocalization, whereas undifferentiated cells demonstrate peri-nuclear and nuclear colocalization. **d** Representative pictures of 10,000 cells acquired using the Amnis FlowSight cytofluorimeter, showing the bright field, nuclear stain (violet), NRF2 (yellow), and merge. The nuclear internalization is shown as percent of cells obtained by three independent experiments. **e** mRNA expression of Notch and Wnt pathway targets (Axin2, Myc, Sox9, Hes1, Hnf1a, Hnf4a, Hnf6), and hepatic maturation genes (albumin, CYP3A4, Ggt-1) in BECs/HPCs treated with a siRNA targeting NRF2 or a control siRNA (scrambled) for 24 h. Data in the graph are represented as mean ± SD. Statistical differences were assessed by student’s *t* test. *p < 0.05 vs scrambled; **p < 0.01 vs scrambled; ***p < 0.001 vs scrambled. **f** NRF2 binding to the HMOX1 and NQO1 promoters in primary hepatocytes and BECs/HPCs isolated from mice fed a control chow (L, lean), 3,5-diethoxycarbonyl-1,4-dihydrocollidine diet (C, cholestasis), or methionine-choline deficient diet (S, steatohepatitis). PCR products were detected on 2% agarose gel. Band intensities were quantified with Image J software. Input DNA was used as a control. **g** Quantitative analysis of ChIP experiments. Fold increase was calculated over their respective primary hepatocytes from lean mice. Data in the graph are represented as mean ± SD of three experiments. Statistical differences were assessed by student’s *t* test. **p < 0.01 vs primary hepatocytes; ***p < 0.001 vs primary hepatocytes.

To identify the role of redox-modulated transcription factors in HPCs, we compared progenitors with differentiated cells. For the purpose of characterizing the main redox-dependent mechanisms that initiate HPCs differentiation, we studied publicly available gene expression data sets from undifferentiated liver stem cells (or hepatoblasts) to differentiated hepatocytes/cholangiocytes using Gene Expression Omnibus 2R and gene set enrichment analysis [GSEA; GEO accession numbers GSE7038^20^ and GSE28891^21^]. Enrichment scores of data sets of undifferentiated versus differentiated cells revealed upregulation of metabolism pathways and downregulation of pathways related to redox homeostasis with the differentiation process (Fig. 2a, Supplementary Fig. 3a, b). This result suggests that the modification of the cellular redox balance towards a pro-oxidative status is associated with the process of HPCs differentiation. In all data sets, analysis of gene ontology terms that were significantly (GSEA: family wise error rate *P* < 0.05) downregulated in differentiated cells demonstrated links with antioxidant function. Particularly referring to transcription factors modulated by reactive species, most of these were characterized as NRF2 target genes (Fig. 2b, Supplementary Table 1).

The link between NRF2 activation and primary BECs/HPCs quiescence/activation was then investigated in BECs/HPCs isolated from lean animals and rodent models of BEC/HPC activation. Particularly, nuclear translocation of NRF2 was found in quiescent BECs/HPCs, whereas it was detected mostly in the cytoplasm of activated cells (Fig. 2c, d). To highlight the key role of NRF2 in determining HPCs quiescence/activation, we used a specific siRNA for gene silencing (Supplementary Fig. 4). Interestingly, NRF2 silencing promoted the expression of both Notch and Wnt-downstream genes, as well as albumin, cytochrome 3A4 (CYP3A4), and gamma-glutamyl transpeptidase 1 (GGT1) genes, markers of hepatic maturation (Fig. 2e). To confirm the role of NRF2 activation on BECs/HPCs, we performed chromatin Immunoprecipitation (ChIP) assays to detect the recruitment of NRF2 to endogenous *HMOX1* and *NQO1* (two NRF2 target genes) promoters in primary hepatocytes and BECs/HPCs isolated from lean animals and rodent models of BEC/HPC activation. The binding of NRF2 to the *HMOX1* and *NQO1* promoters resulted higher in BECs/HPCs rather than mature hepatocytes isolated from lean mice. Interestingly, an increase of NRF2 binding in primary hepatocytes but a marked reduction in BECs/HPCs from models of cholestasis and steatohepatitis was observed (Fig. 2f, g). Of note, NRF2 protein amount was higher in primary hepatocytes—but not BECs/HPCs—from models of liver injury rather than lean mice (Supplementary Fig. 5).

We further studied the localization of NRF2 in the liver of animal models. In healthy liver, NRF2 was detected in the nucleus of cells within the canals of Hering; nevertheless, in mouse models of biliary and hepatocellular regeneration, NRF2 was found in the nucleus (and in the cytoplasm) of parenchymal cells—but not in the cells located in the portal tract, especially those within the bile ductules (Supplementary Fig. 6). Double immunostaining revealed the intranuclear localization of NRF2 in CK19-positive cells in the liver of lean animals; on the contrary, NRF2 was detected in hepatocytes—but not in the nucleus of CK19-positive cells—during steatohepatitis (Supplementary Figs. 7, 8).

**Fig. 3:**
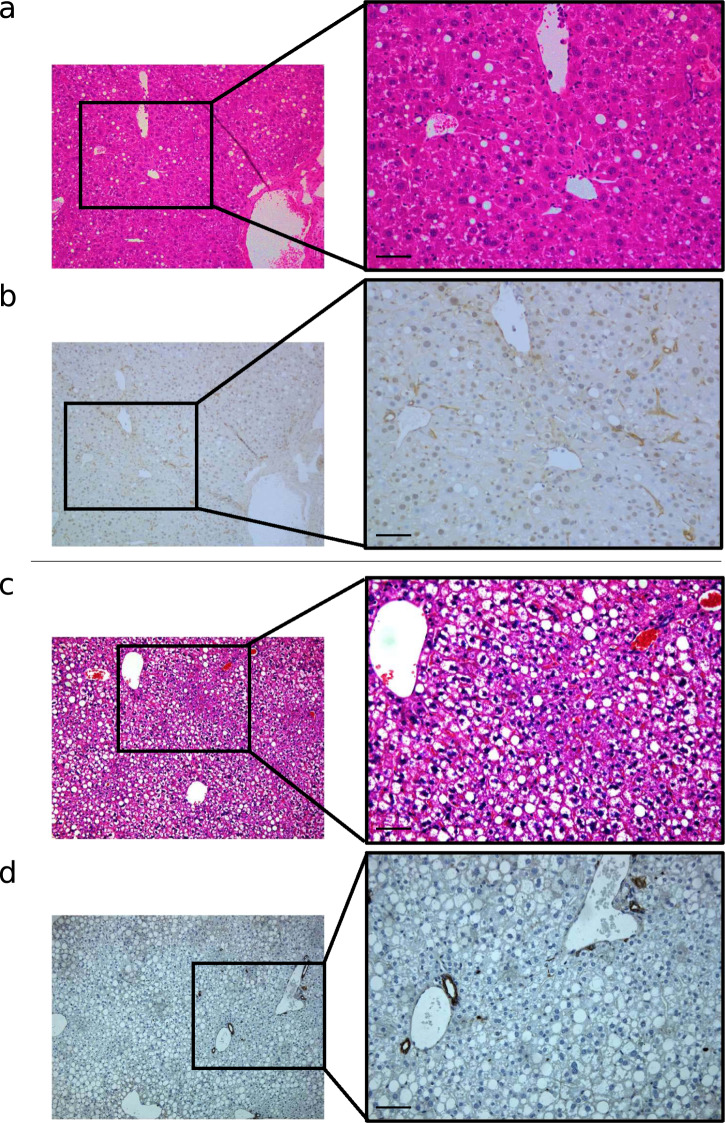
Activation of NRF2 in the hepatic niche impairs ductular reaction in injured liver. Representative images showing hematoxylin/eosin staining (**a**, **c**) or immunohistochemical detection of CK19 (**b**, **d**) in the liver of mice fed a methionine-choline deficient diet (MCD) subjected to the infusion of 5 × 10^9^ plaque forming unit (pfu) of Ad-m-KEAP1-shRNA (an adenovirus expressing shRNA for silencing of mouse Keap1) (**c**, **d**), or (Ad-U6-RNAi (a control adenovirus expressing a scrambled shRNA) (**a**, **b**) in the biliary tract. Of note, steatohepatitis was more severe in the group of mice treated with Ad-m-KEAP1-shRNA rather than Ad-U6-RNAi. Immunohistochemistry was performed in four different experiments using the 3,3’-diaminobenzidine method with hematoxylin counterstaining (left: magnification ×100; right: magnification ×200).

To confirm the role of NRF2 in HPC biology, we studied the ductular reaction in mice fed the methionine and choline-deficient (MCD) diet undergoing activation of NRF2 in the canals of Hering. To this, we administered an adenovirus expressing shRNA for silencing of mouse Kelch-like ECH-associated protein 1 (Keap1, a repressor of NRF2), or a scrambled shRNA as control, within the biliary tract. Validation of the model is reported in Supplementary Fig. 9. As shown in Fig. 3, ductular reaction occurred in controls, but not in animals subjected to Keap1 inhibition (and consequent NRF2 activation) in the biliary tract.

Taken together, these data strongly suggest that NRF2 is constitutively activated in HPCs to maintain stemness, whereas it is inhibited in case of HPC activation.

### NRF2 inhibition increases the transplantation efficiency of human hepatic progenitor-like cells

To establish the key role of NRF2 in HPC modulation, we aimed to verify whether its inhibition in vitro would improve the transplantation efficiency of HPCs in vivo. To track HPCs in a mouse model, we moved to HepaRG cells, a human immortalized liver cell line able to trans-differentiate toward bipotent progenitor cells^22^. Since the effect of a specific siRNA to NRF2 silencing lasted after 48 h, the chemical compound ARE expression modulator 1 (AEM1)—which does not alter NRF2 expression (Supplementary Fig. 10)—was used to inhibit the downstream effect of this transcription factor^23^.

**Fig. 4:**
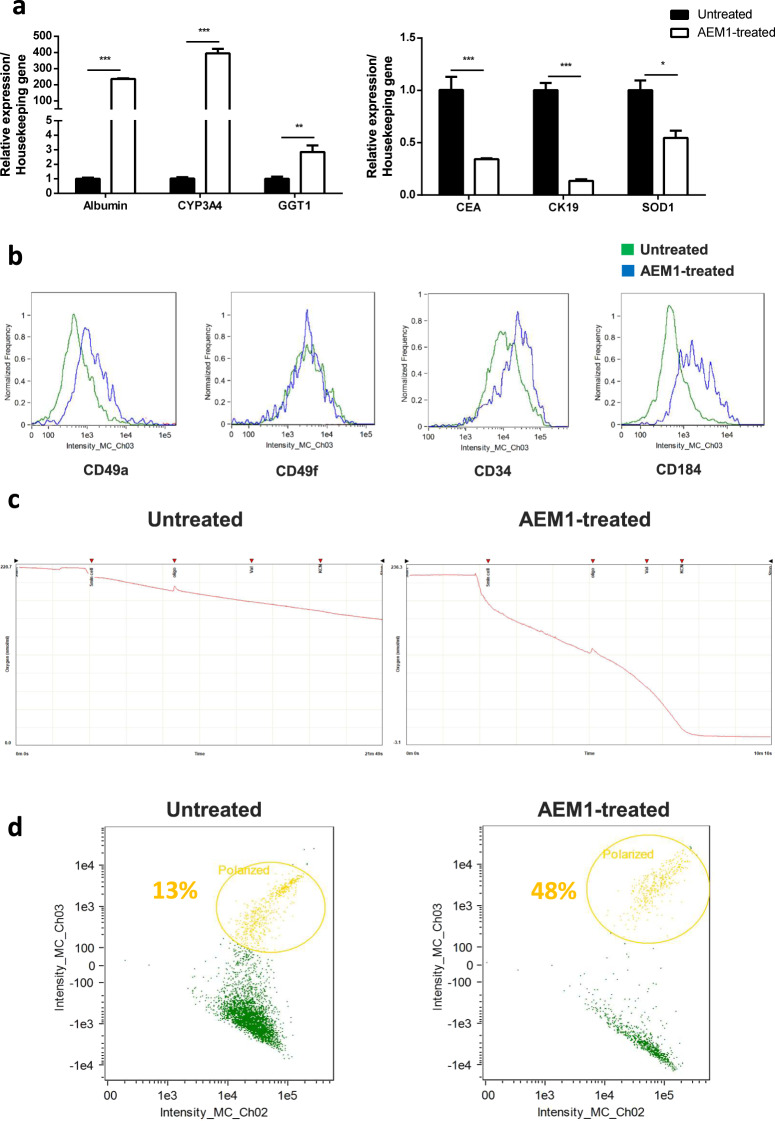
Inhibition of NRF2 via AEM1 treatment induces HepaRG differentiation. **a** mRNA expression of genes characteristic of mature hepatocytes (left) or of indifferentiated cells (right) in untreated or AEM1-treated HepaRG cells after 2 weeks. Data in the graph are represented as mean ± SD of three independent experiments. Statistical differences were assessed by student’s *t* test. *p < 0.05 vs untreated; **p < 0.01 vs untreated; ***p < 0.001 vs untreated. **b** Flow cytometry phenotype of untreated or AEM1-treated HepaRG cells after 2 weeks. **c** Representative recordings of oxygen consumption rate (nmolO_2_/min/mln cell) in untreated or AEM1-treated HepaRG cells after 2 weeks. **d** Mitochondrial membrane potential measured in untreated or AEM1-treated HepaRG cells after 2 weeks via the JC-10 assay.

AEM1 treatment in HepaRG cells resulted in increased expression of albumin, CYP3A4, and GGT1 genes, which are characteristic of mature hepatocytes, whereas the expression of carcinoembryonic antigen (CEA) and cytokeratin 19 (CK19) genes—typical of immature cells—was reduced (Fig. 4a). Furthermore, when compared with untreated HepaRG, AEM1-treated cells showed increased expression of CD49a and CD49f—which are markers of hepatocytes and cholangiocytes, respectively—CD34 and CD184, both markers of stem and progenitor cells (Fig. 4b). Since the differentiation process of stem/progenitor cells is characterized by the increase of mitochondrial metabolism, to further confirm the commitment of HepaRG cells following AEM1 treatment we evaluated both mitochondrial respiration (oxygen consumption) and membrane potential, which were higher AEM1-treated compared with untreated HepaRG cells (Fig. 4c, d). Of note, AEM1 treatment was not associated with changes in the activation of phosphoinositide-3 kinase and extracellular Signal regulated kinases (ERK) pathways (Supplementary Fig. 10). In summary, these results suggest that the inhibition of NRF2 by AEM1 treatment induces the trans-differentiation of HepaRG cells.

**Fig. 5:**
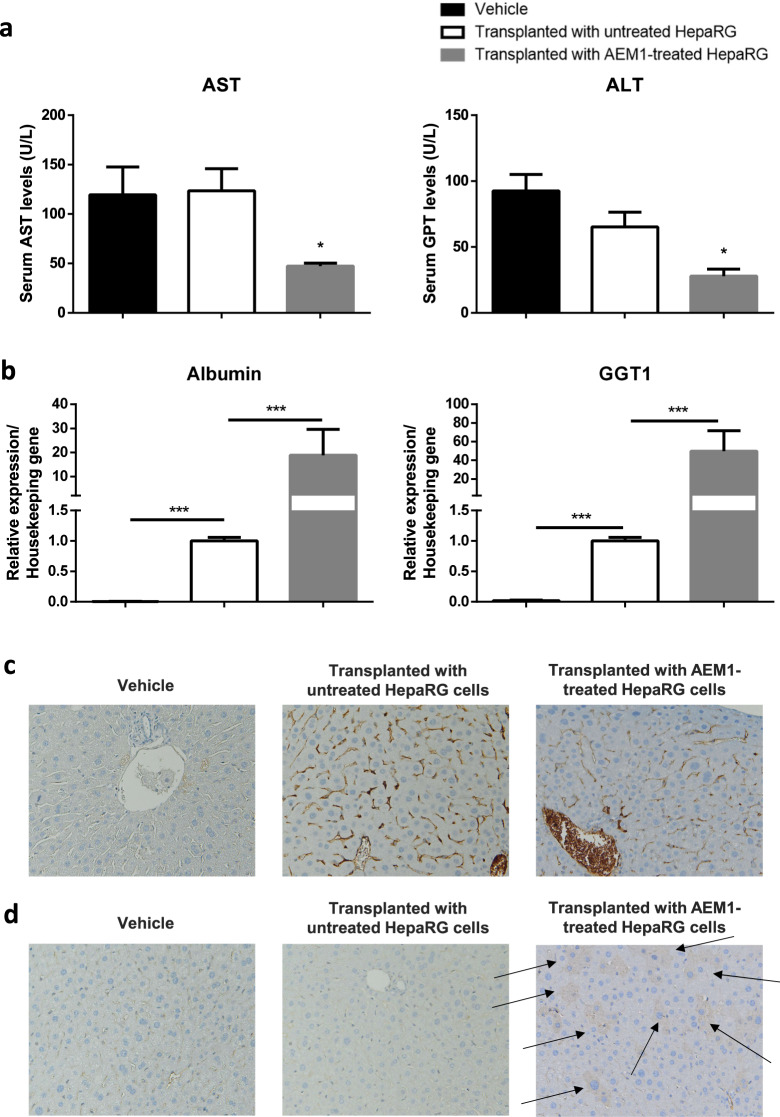
Transplanted AEM1-treated HepaRG cells allow recovery from liver injury. **a** Serum aspartate (AST) and alanine aminotransferase (ALT) levels from SCID Beige mice treated with Jo2 mAb once weekly for 4 consecutive weeks after transplantation. Data in the graph are represented as mean ± SD of four independent experiments. Statistical differences were assessed by one-way ANOVA and Tukey as post hoc test. *p < 0.05 vs untrasplanted and vs trasplanted with untreated HepaRG. **b** mRNA expression of human albumin and gamma-glutamyl transpeptidase 1 (GGT1) in the liver of SCID beige mice treated with Jo2 mAb once weekly for 4 consecutive weeks after transplantation. Data in the graph are represented as mean ± SD of four independent experiments. Statistical differences were assessed by one-way ANOVA and Tukey as post hoc test. ***p < 0.001. **c** Representative images showing immunohistochemical detection of human Cytokeratin 19 in the liver of SCID Beige mice treated with Jo2 mAb once weekly for 4 consecutive weeks after transplantation (magnification ×200). **d** Representative images showing immunohistochemical detection of human Glypican 3 in the liver of SCID beige mice treated with Jo2 mAb once weekly for 4 consecutive weeks after transplantation (magnification ×200).

SCID beige mice were used as mouse model for HepaRG transplant recipient. Both untreated and AEM1-treated HepaRG cells were injected intrasplenically in mice; starting from the following day, anti-Fas mAb (Jo2) was intraperitoneally administered once weekly for 4 weeks, to induce chronic liver injury by selective hepatocellular apoptosis^24^. Cell transplantation was initially verified by tracking HepaRG cells in vivo with the fluorescent probe DiIC_18_(7) (1,1’-Dioctadecyl-3,3,3’,3’-Tetramethylindotricarbocyanine Iodide) (DIR, Supplementary Fig. 11a–c). To evaluate hepatocellular necrosis, serum levels of both alanine (ALT) and aspartate (AST) aminotransferase were measured. Liver injury caused by Jo2 injection was characterized by increased AST and ALT levels in control mice; the serum level of both transaminases was similar in mice transplanted with untreated HepaRG cells, whereas a significant reduction was observed in those transplanted with AEM1-treated HepaRG cells (Fig. 5a). The increased expression of both human ALBUMIN and GGT1 in mice transplanted with AEM1-treated HepaRG cells, compared with those transplanted with untreated cells, indicated a functional recovery after chronic liver injury (Fig. 5b). Finally, immunohistochemical staining with specific anti-human CK19, CK7, Glypican 3, and albumin showed that HepaRG cells engrafted the mouse liver, but AEM1-treated cells only were able to differentiate and replace the damaged parenchyma (Fig. 5c, d; Supplementary Fig. 12).

## Discussion

This study demonstrates the role played by the redox-dependent transcription factor nuclear factor erythroid-derived 2-like 2 (NRF2) in modulating the activation/differentiation process of HPCs, suggesting its therapeutic potential.

BEC/HPC activation occurs in rodent models of both cholestatic and hepatocellular liver injury, replacing the damaged tissue^4^. The present data evidence that oxidative stress is associated with the activation and proliferation of HPCs in such models of chronic liver injury. A bioinformatics analysis based on previous transcriptomics data sets pointed out changes in the gene level of several antioxidant enzymes whose expression is regulated by NRF2. Indeed, NRF2 is a redox-sensitive transcription factor that activates cytoprotective signaling pathways against oxidative, inflammatory, and pro-apoptotic damage through the induction of several genes encoding phase II detoxifying enzymes and antioxidants^10^. The activation of NRF2 occurs in viral, alcoholic, non-alcoholic, and toxic hepatitis, playing a crucial role in the protection of hepatocellular elements from oxidative damage^25^. It was recently reported that NRF2 activation in hepatocytes induces a ductular reaction through the release of high-mobility group box 1, even though the specific deletion of NRF2 in hepatocytes is not sufficient to inhibit ductular reaction^26^. Our data show that NRF2 is activated in the hepatic parenchyma of chronic disease. On the contrary, focusing on the bile ductules, where the progenitor cell niches are located, NRF2 appears constitutively activated in healthy liver but de-activates in models of hepatic injury. These observations were confirmed by proving that, in primary BECs/HPCs, the NRF2 binding to target gene promoters is higher in healthy than injured liver. Furthermore, NRF2 activation in the biliary tract of mice developing steatohepatitis results in the repression of any ductular reaction.

There is a growing evidence on the novel function of the NRF2 pathway in stem cell proliferation and differentiation^27^. For instance, the main role played by the NRF2 system in maintaining intestinal stem cells homeostasis has been well-characterized, as this transcription factor is constitutively active to maintain stemness^28^. Furthermore, the NRF2 pathway is relevant in determining pluripotent stem cells reprogramming^29^, neural stem cell fate^30^, and differentiation of mesenchymal stem cells toward osteoblasts^31^. Our study demonstrates the involvement of NRF2 in the modulation of HPC homeostasis, adding an important piece to the puzzle of the redox contribution in stem cell biology^32^.

Exposure of BECs/HPCs to reactive species induces specific changes in the cell cycle characteristic of replicative events, and triggers the expression of genes downstream the Notch and the Wnt pathways, typical of committed cells. On the other side, a pro-oxidant environment induces the decrease of EpCAM in BECs/HPCs. Of note, EpCAM expression declines as progenitor cells differentiate, suggesting that the level of cellular differentiation may also depend on EpCAM level^33^. It is then conceivable that, in the quiescent state, NRF2 activation contributes to the maintenance of low levels of oxidants in the hepatic niche, preventing HPC senescence. On the contrary, during chronic liver disease NRF2 de-activates to allow a pro-oxidant environment in the niche and to promote the activation of progenitors.

Our experiments demonstrate that both the genetic and the pharmacological inhibition of NRF2 promote the proliferation and differentiation of BECs/HPCs. Indeed, NRF2 inhibition is associated with the higher expression of Notch and Wnt-downstream genes, the two main transcription factors involved in the commitment of liver progenitors toward cholangiocytes and hepatocytes, respectively^4^. Further, NRF2 inhibition drives the trans-differentiation of HepaRG cells, a human liver-derived cell line that shows phenotypical markers of hepatocyte-like and cholangiocyte-like cells^22^.

Compared with differentiated cells, quiescent stem cells in the niches are characterized by low levels of reactive species and a reduced rate of oxidative phosphorylation. Several studies demonstrate the different metabolic phenotype between stem and differentiated cells, underlining the shift from anaerobic to aerobic metabolism and the increased production of reactive species during the differentiation process^34,35^. Particularly, haematopoietic stem cells residing in the bone marrow niches are surrounded by a hypoxic microenvironment, producing energy by high glycolysis rate rather than via oxidative phosphorylation^36^. The present data demonstrate that NRF2 inhibition in human hepatic progenitor-like cells is associated with an increase in mitochondrial oxygen consumption and membrane potential.

HPCs have been suggested as an important source for liver cell transplantation, even though these cells do not regenerate significant quantity of liver parenchyma^37,38^. Several investigations so far have described that the transplantation efficiency of HPCs is too low to produce enough functional mature hepatocytes^39,40^. In vitro models of HPC maturation, other than being useful for elucidating the differentiation mechanism of progenitors into mature liver cells, might improve the efficiency and biosafety profile of possible clinical applications for liver stem cell transplantation^41^. We then verified whether NRF2 inhibition would effectively enhance the effects of transplantation in terms of structural and functional recovery during chronic liver damage. To this, the human HepaRG cell line was transplanted in SCID beige mice. Our data demonstrate that NRF2 inhibition in HepaRG cells favors engraftment and repopulation of damaged cells by transplanted elements. Furthermore, NRF2 inhibition is associated with an in vivo functional outcome—i.e., the production of albumin and GGT—in transplanted HepaRG cells.

In conclusion, this study demonstrates that NRF2 is crucial to determine the HPC fate. Furthermore, the inhibition of NRF2 at a pre-transplant stage in HPCs, other than proving the role of this transcription factor for the activation/differentiation of progenitors, is promising in terms of cell engraftment and differentiation improvement following transplantation. These findings pave the way for further investigation aimed at achieving clinical outcomes.

## Methods

### Mouse models of HPC activation

Animal experimental protocols were approved by the University of Foggia ethics committee and conducted in accordance with the guidelines of the Italian Ministry of Health (D.L. 26/2014) and the European Parliamentary directive 2010/63/EU.

Two validated dietary mouse models of HPC activation on a C57BL6/J background were used^4,42^:

(a) Mice fed an MCD diet for up to 2 weeks, which induces hepatocellular injury and HPC-mediated hepatocyte regeneration.

(b) C57B mice treated with 0.1% (wt/wt) 3,5-diethoxycarbonyl-1,4-dihydrocollidine diet in Purina 5015 mouse chow for up to 18 d. This induces biliary injury and regeneration.

### Cell cultures

#### Primary hepatocyte isolation and culture

Hepatocytes were isolated from non-treated animals and rodent models of BEC/HPC activation using a modified perfusion technique and cultured^43^. In brief, Liver Perfusion Medium (Thermo Fisher Scientific, 17701038) for 5 min followed by Liver Digest Medium (Thermo Fisher Scientific, 17703034) for 10 min were used for perfusion. The liver was subsequently excised, and the capsule disrupted to yield a cell suspension in Liver Perfusion Medium, which was passed through a Falcon 100 μm cell strainer (Corning, 352360). Hepatocytes were then pelleted by centrifugation at 135 G for 1 min, separated from a non-parenchymal cell-rich fraction, and resuspended in Williams E Medium (Thermo Fisher Scientific, 12551032) with 5% FBS. Cells were underlayered with a discontinuous Percoll gradient (1.06, 1.08 and 1.12 mg/ml Percoll in PBS) and spun at 750 G for 20 min at 20 °C. Cells collected between the 1.08 and 1.12 mg/ml Percoll layers were then harvested and resuspended in Williams medium.

#### FACS based BEC/HPC isolation and culture

Primary BECs/HPCs were extracted by lean C57Bl6 mice and rodent models of BEC/HPC activation. Fluorescence-activated cell sorting (FACS) separation of BECs/HPCs was performed using EpCAM+/CD24+/CD133+/CD31−/CD45−/Ter119− sorting (Supplementary Fig. 13). FACS characteristics of BECs/HPCs from uninjured and injured liver were described previously^5^. After isolation by medium containing collagenase B and DNAse I, cells were centrifuged through a discontinuous Percoll gradient and cultured as previously described^4^.

#### HepaRG culture

The human cell line HepaRG was purchased by Merck Millipore (MMHPR116). Undifferentiated HepaRG cells exhibit a fibroblast-like morphology, and the differentiation process induces both hepatocyte-like and biliary-like epithelial phenotypes at confluence, indicating bipotent progenitor features^22,44^. HepaRG cells were seeded at 27000 cell/cm^2^ confluence in a base medium composed by William’s E Medium + GlutaMAX (Gibco, 3255-020) supplemented with 10% FBS (Sigma-Aldrich, F7524), 100 U/ml penicillin (Sigma-Aldrich, 13752), and 100 μg/ml streptomycin (Sigma-Aldrich, P4333).

### Cell treatment for redox manipulation

BECs/HPCs were exposed to pro-oxidants and antioxidants added to the culture medium. Primary BECs/HPCs were treated with different concentrations (from 1 nM to 100 µM) of H_2_O_2_, t-BuOOH (Sigma-Aldrich, BSO (Sigma-Aldrich, B2515), or antimycin A (Sigma-Aldrich, A8674). To render cells more reduced, the medium was supplemented with NAC (Sigma-Aldrich, A7250). Acute toxicity of each substance was studied after 24 h by the microculture tetrazolium assay^45^, and the highest non-toxic dosage was used in subsequent experiments.

### Cell adhesion and spreading assays

For the adhesion assay, cells were cultured with pro-oxidants or NAC overnight. In all, 5 × 10^4^ BECs/HPCs were seeded in 96-well plates precoated with laminin (20 μg/ml) for 30 min. Cells were washed, and adherent cells were fixed in 5% glutaraldehyde (Sigma-Aldrich, 340855) and stained with 0.1% crystal violet. After dye solubilization in 10% acetic acid, absorbance was measured at 570 nm.

To assay spreading, we used Oris^TM^ Cell Migration Assay kit (Platypus Technologies, CMA1.101) as per the manufacturer’s protocol. The area of migrating cells after 24 h was calculated using Image J software. The results of all these experiments were obtained from at least four separate experiments.

### NRF2 silencing

Primary BECs/HPCs were seeded 0.4–1.6 × 10^5^ cells per well in a 24-well plate, transiently transfected with 37.5 ng of a pre-designed siRNA directed against mouse NRF2 (Mm_Nfe2l2_1 FlexiTube siRNA, Qiagen, NM_010902), using the HiPerFect Transfection Reagent (Qiagen, 301704). According to the manufacturer procedure, siRNA was diluted in 100 µl culture medium; subsequently, 3 µl of HiPerFect Transfection Reagent were added to the diluted siRNA, to form transfection complexes which were loaded in the wells for 24 h. The following day, cells were used for gene analysis by realt-ime PCR.

### AEM1 treatment

AEM1 was used to inhibit Nrf2 binding to antioxidant response elements (AREs). AEM1 does not induce modifications in the expression of both Nrf2 and Keap1 protein^23^. AEM1 (Sigma-Aldrich, SML-1556) was used at the final concentration of 1 μM dissolved in growth medium.

### Gene expression analyses

To study the levels of genes expressed in both BECs/HPCs and HepaRG cells, RNA was extracted from 1.0 × 10^6^ cells/sample and converted into cDNA, which was used as template in the following Real Time PCR. To isolate RNA from frozen livers, 30 mg/sample were used. The tissue was included in Lysis buffer (+1% β-mercapthoethanol) and homogenized by potter.

RNA extraction was performed by the “Pure Link RNA Mini kit” (Thermo Fisher, 12183025), according to the manufacturer’s protocol. RNA concentration was determined by spectrophotometer method at Nanodrop, measuring absorbance at λ = 260 nm. *A*_260_/*A*_280_ > 2 was evaluated to guarantee protein-free samples.

Reverse transcription was performed by high-capacity cDNA Reverse Transcription Kit (Applied Biosystems, 4368814), and SYBR Green (Biorad, 172-5271) was used as fluorescent probe. Murine and Human *actin* were chosen as housekeeping genes. The sequences of forward and reverse primers of all the genes studied are provided in the Supplementary information (Supplementary Table 2), except than primers used to amplify mouse-specific NRF2, albumin, HES1, HES5, HNF1a, HNF4a, SOX9, MYC, and AXIN2 genes, which were purchased by Qiagen (QuantiTect Primers Assay), and primers used to amplify mouse-specific CD326 (Epcam), CYP3A4, and Ggt-1, which were purchased by Bio-Rad (PrimePCR™ SYBR® Green Assay).

### ChIP assay

ChIP assay was performed using the EZ-ChIP assay kit (Millipore, Billerica, MA). In brief, both primary hepatocytes and BECs/HPCs were cross-linked with formaldehyde, and chromatin fragmentation was performed according to the kit procedure. Diluted soluble chromatin solution was incubated with anti-NRF2 primary antibody (Abcam, ab31163) for 18 h at 4 °C with rotation. Following incubation with protein A/G agarose beads, the bound products were washed, and DNA was eluted. DNA was subjected to PCR with primers encompassing the functional AREs located upstream of transcriptional start site of HMOX1 and NQO1 to determine the binding of NRF2 in ChIP assays.

### Confocal microscopy

In all, 1.5 × 10^5^ cells/well were seeded on the glass coverslip in a 24-multi wells plate. The day after, cells were washed three times with PBS, fixed with 4% paraformaldehyde for 10 min at RT, and washed two times with PBS. Cells were firstly permeabilised with PBS + 0.1% X-100 Triton (Fluka, 93418) for 10 min, then blocking buffer (3% BSA (Sigma, A7906) + 0.3 M Glycine (Sigma, G-7126-500-50) was added for 30 min at RT. Subsequently, cells were treated for 1 h and a half at RT with anti-NRF2 primary antibody (Abcam, ab31163), and then washed three times with PBS. Cells were then labeled with AlexaFluor 488-conjugated secondary antibody (Abcam, ab150073) in the dark for 1 h at RT. Nuclei were counterstained with DAPI included in the mounting medium (Abcam, ab104139). Cells were analyzed by a Nikon Eclipse Ti-E confocal microscope.

### Flow cytometry

Phenotype changes, cell cycle, and mitochondrial membrane potential were investigated by flow cytometry analysis, using the FlowSight Cytometer (Amnis, Merck Millipore) and the IDEAS Software.

To study the cellular phenotype, PE-labeled antibodies against CD34 (1:50, 130-081-002), CD184 (CXCR4; 1:10, 130-098-354), CD49a (1:50, 130-101-397), CD49f (1:50, 130-097-246), CD326 (EpCAM; 1:50, 130-091-253), rat IgG1ĸ isotype control (1:10, 130-102-645), and FITC-labeled CK (1:11, 130-080-101) were purchased by Miltenyi Biotec. In brief, 1.0 × 10^6^ cells/sample were resuspended in PBS and left 10 min at 4 °C in the dark; then, cells were centrifuged at 300 × *g* for 10 min, and washed twice with PBS. Finally, samples were resuspended in PBS and analyzed by flow cytometry. Cells labeled with anti-CK were permeabilised with PBS + 0.1% X-100 Triton before staining.

To study the cell cycle, cells were washed with PBS and centrifuged at 300 × *g* for 3 min; pellet was resuspended in the medium and cells counted. The supernatant was discarded and cold EtOH was added to the pellet; the samples were then vortexed and preserved at −20 °C overnight. The day after, cells were centrifuged at 300 × *g* for 3 min and stained with 5 μM DRAQ5 (BioLegend, 424101) for 15 min, at RT.

Mitochondrial membrane potential was analyzed using JC-10 (Abcam, ab112133) as a probe. In all, 5.0 × 10^5^ cells were incubated with JC-10 for 15 min at 37 °C in the dark before flow cytometry analysis.

Apoptosis was quantified by using annexin V (AV)/7-amino-actinomycin D detection kit (Beckman Coulter Inc, Indianapolis, IN, USA) according to the manufacturer’s instructions.

### Respirometry on HepaRG cells

In all, 5.0 × 10^6^ HepaRG cells were washed with PBS and resuspended in 10 mM KH_2_PO_4_, 27 mM KCl, 1 mM MgCl_2_, 40 mM HEPES, 0.5 mM EGTA buffer (pH 7.1), and assayed for O_2_ consumption by the Oxygraph Plus System (Hansatech Instruments) at 37 °C under continuous stirring. Oligomycin (8 µg/ml) was added followed by the addition of valinomycin (2 µg/ml) after 5 min. The rates of oxygen consumption were corrected for 3 mM KCN-insensitive respiration and normalized to the cell number. Each experiment was repeated in triplicate.

### DIR labeling

DIR (Thermo Fisher, D12731) is a near IR fluorescent, lipophilic dye, quite photostable when incorporated into membranes: once applied to cells, the dye diffuses within the plasma membrane. This dye is useful for in vivo cell tracking; however, we initially tested its toxicity in vitro. To evaluate the highest non-toxic concentration, cells were labeled with DIR. At two different final concentrations (5 and 2.5 µM). 1.0 × 10^5^ cells/sample were incubated for 20 min with DIR at 37 °C in the dark. When the incubation time ended, cells were washed with PBS and counted with Trypan Blue (Sigma, T8154) to measure cell viability. The final concentration of 2.5 µM was established as non-toxic for HepaRG cells.

### Bioinformatic analysis

Microarray data from undifferentiated HPCs and differentiated hepatocytes/cholangiocytes^20,21^ were analyzed for transcript expression using the Kyoto encyclopaedia of genes and genomes (KEGG), gene ontology (GO), or GSEA analysis. Raw microarray data are also publicly available on GEO under the accession numbers GSE7038 and GSE28891. Venn diagram was drawn with Intervene software^46^.

### HepaRG cell transplantation

SCID Beige mice (Charles River) were chosen as animal model. These mice are characterized by severe immunodeficiency affecting B and T lymphocytes, and natural killer cells.

To evaluate the intrahepatic diffusion of cells after transplant, 1.0 × 10^6^ untreated or AEM1-treated cells were labeled with DIR immediately before intrasplenic injection^47^. Migration of transplanted cells from the spleen to the liver was detected after 30 min and after 24 h by the In vivo F-PRO (Bruker). The presence of transplanted HepaRG cells was also evaluated in the explanted spleen and liver after 4 weeks.

The day after HepaRG cell transplantation, liver injury was induced by administration of an anti-Fas monoclonal Ab (Jo2/CD95, BD Bioscences), once weekly for 4 weeks. Mice were divided in the following groups:

- Controls, transplanted with vehicle (*n* = 4);
- Transplanted with untreated HepaRG cells (*n* = 4);
- Transplanted with AEM1-treated HepaRG cells (*n* = 4).

At the end of the fourth week, animals were killed, the blood drawn, and the livers excised to be frozen at −80 °C; some liver samples were fixed in 4% formalin for histopatology and immunohistochemistry. Hepatocellular necrosis was assessed by measuring the serum level of AST and ALT, respectively.

### Biliary administration of adenovirus

Infusion of adenovirus via the biliary tract was performed placing a cannula into the bile duct^48^. In brief, bile duct cannulation was performed under an operating microscope with ×4–6.4 magnification. Following a midline abdominal incision, the liver was exposed, the falciform ligamentum anterior was cut, and the median liver lobe was displaced to expose the gallbladder, cystic duct, hepatic ducts, and common bile duct. A 10 mm-long BD Intramedic™ polyethylene tube (Inner Diameter 0.011 in., Outer Diameter 0.024 In., Thermo Fisher Scientific, BD 427400), connected to a Dow Corning™ Silastic™ Laboratory silicone tube (Inner Diameter 0.02 in., Outer Diameter 0.037 in., Thermo Fisher Scientific, Dow Corning™ 2415500) was inserted into the distal site of the gallbladder, and moved up to the origin of the cystic duct. To avoid antegrade outflow, the common bile duct was flushed with saline and clamped. In all, 5 × 10^9^ plaque forming unit (pfu) of Ad-m-KEAP1-shRNA, an Adenovirus expressing shRNA for silencing of mouse Keap1 (Vector Biolabs, shADV-262778), or Ad-U6-RNAi, a control adenovirus expressing a scrambled shRNA (Vector Biolabs, 1640), diluted in 100 μL of serum-free DMEM, were injected in C57Bl6/J mice fed a MCD diet for up to 2 weeks with an approximate flow rate of 10 μl/min.

### Immunohistochemistry

Immunohistochemical analysis on 4-μm serial sections was performed by using Ventana Benchmark® XT autostainer and standard linked streptavidin-biotin horseradish peroxidase technique (LSAB-HRP), according to the best protocol for each antibody used in our laboratory: primary mouse polyclonal antibody anti-human CK19 diluted 1:100 in PBS (Ventana, 760-4281); primary mouse monoclonal antibody anti-human CK7 diluted 1:100 in PBS (OV-TL 12/30, Cell Marque, CMC30729050); primary mouse monoclonal antibody anti-human Glypcan3 diluted 1:300 in PBS (Ventana, 790-4564); primary mouse monoclonal antibody anti-human albumin diluted 1:150 in PBS (MyBioSource, MBS766511) and incubated overnight. Negative control slides without primary antibodies were included for each staining. A similar protocol was used for HNE (1:200; Abcam, ab46545) and NRF2 (1:150; Abcam, ab31163) analysis. Double immunostaining was performed using CK19 and NRF2 antibodies. Primary antibodies were revealed by automated staining device (Leica BOND RX) using standard LSAB-HRP and linked streptavidin-biotin alkaline phosphatase (LSAB-AP) techniques performed at the same time. The results of the immunohistochemical staining were evaluated separately by two observers. In each tissue section, 10 representative high-power fields were analyzed at optical microscope (Zeiss Axioscope). Digital image analysis was performed by the Image Pro-Premier Software version 9.1 (Media Cybernetics).

### Western blot analysis

In all, 30 μg proteins from cell homogenates were loaded in a 3–8% sodium dodecyl sulphate–polyacrylamide gel electrophoresis and transferred to a nitrocellulose membrane, blocked for 1.5 h using 5% nonfat dry milk in TBS-t and incubated with rabbit anti-NRF2 primary antibody (1:1000; Abcam, ab137550), rabbit anti-phospho-Akt (Ser473) (1:1000; Abcam ab81283), rabbit anti-Akt (1:1000; Abcam, ab126811), rabbit anti-phospho-ERK1/2 (Thr202 + Tyr204) (1:250; Abcam, ab214362), rabbit anti-ERK1/2 (1:500; Abcam, ab196883), rabbit anti-β-actin (1:2000; Abcam, ab8227) overnight at 4 °C. Then, the membrane was incubated for 1.5 h with a goat HRP-conjugated anti-rabbit secondary antibody (1:2000; Abcam, ab205718). Bands were detected by the Clarity™ Western ECL Blotting Substrate using a ChemiDoc MP system (Bio-Rad Laboratories Inc) and quantified by the Image Lab™ Software.

### Western blot analysis of hepatic oxidized proteins

Analysis of oxidized proteins was performed by western blot in liver homogenates using an Oxyblot kit (Millipore Bioscience Research Reagents, Temecula, CA). The same amounts of liver proteins (35 μg) were reacted with dinitrophenylhydrazine (DNP) for 20 min, followed by neutralization with a solution containing glycerol and 2-mercaptoethanol, resolved in 12.5% sodium dodecyl sulphate–polyacrylamide gel electrophoresis, transferred to a nitrocellulose membrane, blocked with nonfat milk, and incubated with a rabbit anti-DNP antibody (1: 150) at 4 °C overnight. After washing, the membrane was incubated with the secondary antibody (1:300) conjugated to horseradish peroxidase and detected by a chemiluminescence detection kit (Cell Signaling Technology Inc., Danvers, MA). Reactive bands were visualized by the enhanced chemiluminescence method on a VersaDoc Image System (Bio-Rad Laboratories, Hercules, CA).

### Statistical analysis

Data were represented mean ± SEM, with significance determined via Student’s unpaired *t* test or one-way ANOVA if comparing more than two groups. Multiple comparisons used either Turkey’s multiple comparisons test or Dunnett’s multiple comparisons test when appropriate. All statistical tests were performed using GraphPad Prism 6.0 (San Diego, CA). A *p* value of ≤0.05 was considered significant.

### Reporting summary

Further information on research design is available in the Nature Research Reporting Summary linked to this article.

## Supplementary information

Supplementary Information

Reporting Summary

## Data Availability

All relevant data supporting the findings of this study are available within the paper, its supplementary information and from the corresponding authors upon reasonable request. Raw microarray data are publicly available on GEO under the accession numbers GSE7038 and GSE28891.

## References

[1] Allain JE (2002). Immortalization of a primate bipotent epithelial liver stem cell. Proc. Natl. Acad. Sci. USA.

[2] Fellous TG (2009). Locating the stem cell niche and tracing hepatocyte lineages in human liver. Hepatology.

[3] Gouw AS, Clouston AD, Theise ND (2011). Ductular reactions in human liver: diversity at the interface. Hepatology.

[4] Boulter L (2012). Macrophage-derived Wnt opposes Notch signaling to specify hepatic progenitor cell fate in chronic liver disease. Nat. Med..

[5] Lu WY (2015). Hepatic progenitor cells of biliary origin with liver repopulation capacity. Nat. Cell Biol..

[6] Alison MR, Lin WR (2016). Diverse routes to liver regeneration. J. Pathol..

[7] Itoh T, Miyajima A (2014). Liver regeneration by stem/progenitor cells. Hepatology.

[8] Jadeja, R. N., Devkar, R. V. & Nammi, S. Oxidative stress in liver diseases: pathogenesis, prevention, and therapeutics. *Oxid. Med. Cell Longev*. **2017**, 8341286 (2017). 10.1155/2017/8341286 PMC5424478 28529677

[9] Sun S (2018). Advanced oxidation protein products induce S-phase arrest of hepatocytes via the ROS-dependent, beta-catenin-CDK2-mediated pathway. Redox Biol..

[10] Motohashi H, Yamamoto M (2004). Nrf2-Keap1 defines a physiologically important stress response mechanism. Trends Mol. Med..

[11] Liu H, Colavitti R, Rovira II, Finkel T (2005). Redox-dependent transcriptional regulation. Circ. Res..

[12] Sablina AA (2005). The antioxidant function of the p53 tumor suppressor. Nat. Med..

[13] Funato Y, Michiue T, Asashima M, Miki H (2006). The thioredoxin-related redox-regulating protein nucleoredoxin inhibits Wnt-beta-catenin signalling through dishevelled. Nat. Cell Biol..

[14] Miyamoto K (2007). Foxo3a is essential for maintenance of the hematopoietic stem cell pool. Cell Stem Cell.

[15] Chuikov S, Levi BP, Smith ML, Morrison SJ (2010). Prdm16 promotes stem cell maintenance in multiple tissues, partly by regulating oxidative stress. Nat. Cell Biol..

[16] Kajla S (2012). A crucial role for Nox 1 in redox-dependent regulation of Wnt-beta-catenin signaling. FASEB J..

[17] di Bello G, Vendemiale G, Bellanti F (2018). Redox cell signaling and hepatic progenitor cells. Eur. J. Cell Biol..

[18] Tirnitz-Parker JE (2007). Isolation, culture and immortalisation of hepatic oval cells from adult mice fed a choline-deficient, ethionine-supplemented diet. Int. J. Biochem. Cell Biol..

[19] Dolle L, Boulter L, Leclercq IA, van Grunsven LA (2015). Next generation of ALDH substrates and their potential to study maturational lineage biology in stem and progenitor cells. Am. J. Physiol. Gastrointest. Liver Physiol..

[20] Conigliaro A (2008). Isolation and characterization of a murine resident liver stem cell. Cell Death. Differ..

[21] Shin S (2011). Foxl1-Cre-marked adult hepatic progenitors have clonogenic and bilineage differentiation potential. Genes Dev..

[22] Cerec V (2007). Transdifferentiation of hepatocyte-like cells from the human hepatoma HepaRG cell line through bipotent progenitor. Hepatology.

[23] Bollong MJ (2015). A small molecule inhibits deregulated NRF2 transcriptional activity in cancer. ACS Chem. Biol..

[24] Jiang L (2010). Human hepatoma HepaRG cell line engraftment in severe combined immunodeficient x beige mice using mouse-specific anti-Fas antibody. Transplant. Proc..

[25] Tang W, Jiang YF, Ponnusamy M, Diallo M (2014). Role of Nrf2 in chronic liver disease. World J. Gastroenterol..

[26] Khambu B (2018). HMGB1 promotes ductular reaction and tumorigenesis in autophagy-deficient livers. J. Clin. Invest.

[27] Murakami S, Motohashi H (2015). Roles of Nrf2 in cell proliferation and differentiation. Free Radic. Biol. Med..

[28] Hochmuth CE, Biteau B, Bohmann D, Jasper H (2011). Redox regulation by Keap1 and Nrf2 controls intestinal stem cell proliferation in Drosophila. Cell Stem Cell.

[29] Hawkins KE (2016). NRF2 orchestrates the metabolic shift during induced pluripotent stem cell reprogramming. Cell Rep..

[30] Pistollato F, Canovas-Jorda D, Zagoura D, Bal-Price A (2017). Nrf2 pathway activation upon rotenone treatment in human iPSC-derived neural stem cells undergoing differentiation towards neurons and astrocytes. Neurochem. Int..

[31] Tao J (2016). Downregulation of Nrf2 promotes autophagy-dependent osteoblastic differentiation of adipose-derived mesenchymal stem cells. Exp. Cell Res..

[32] Dai X (2020). Nrf2: redox and metabolic regulator of stem cell state and function. Trends Mol. Med..

[33] Dolle L (2015). EpCAM and the biology of hepatic stem/progenitor cells. Am. J. Physiol. Gastrointest. Liver Physiol..

[34] Wanet A, Arnould T, Najimi M, Renard P (2015). Connecting mitochondria, metabolism, and stem cell fate. Stem Cells Dev..

[35] Cliff TS, Dalton S (2017). Metabolic switching and cell fate decisions: implications for pluripotency, reprogramming and development. Curr. Opin. Genet. Dev..

[36] Suda T, Takubo K, Semenza GL (2011). Metabolic regulation of hematopoietic stem cells in the hypoxic niche. Cell Stem Cell.

[37] Espanol-Suner R (2012). Liver progenitor cells yield functional hepatocytes in response to chronic liver injury in mice. Gastroenterology.

[38] Jors S (2015). Lineage fate of ductular reactions in liver injury and carcinogenesis. J. Clin. Invest..

[39] Ichinohe N (2013). Differentiation capacity of hepatic stem/progenitor cells isolated from D-galactosamine-treated rat livers. Hepatology.

[40] Sharma AD (2008). Murine embryonic stem cell-derived hepatic progenitor cells engraft in recipient livers with limited capacity of liver tissue formation. Cell Transplant..

[41] Gridelli B (2012). Efficient human fetal liver cell isolation protocol based on vascular perfusion for liver cell-based therapy and case report on cell transplantation. Liver Transpl..

[42] Raven A (2017). Cholangiocytes act as facultative liver stem cells during impaired hepatocyte regeneration. Nature.

[43] Bellanti F (2018). Synergistic interaction of fatty acids and oxysterols impairs mitochondrial function and limits liver adaptation during nafld progression. Redox Biol..

[44] Aninat C (2006). Expression of cytochromes P450, conjugating enzymes and nuclear receptors in human hepatoma HepaRG cells. Drug Metab. Dispos..

[45] Kouadio JH (2005). Comparative study of cytotoxicity and oxidative stress induced by deoxynivalenol, zearalenone or fumonisin B1 in human intestinal cell line Caco-2. Toxicology.

[46] Khan A, Mathelier A (2017). Intervene: a tool for intersection and visualization of multiple gene or genomic region sets. BMC Bioinformatics.

[47] Ezzat T, Dhar DK, Malago M, Olde Damink SW (2012). Dynamic tracking of stem cells in an acute liver failure model. World J. Gastroenterol..

[48] Peeters MJ (1996). Adenovirus-mediated hepatic gene transfer in mice: comparison of intravascular and biliary administration. Hum. Gene Ther..

