# Supplementary material for: Inhibition of nuclear factor (erythroid-derived 2)-like 2 promotes hepatic progenitor cell activation and differentiation
Source: NPJ Regen Med. 2021 May 26;6:28. doi: 10.1038/s41536-021-00137-z (PMC8155039; doi:10.1038/s41536-021-00137-z)
Supplement: Supplementary file 1 — Supplementary Information [file 41536_2021_137_MOESM1_ESM.pdf]

Supplementary Information

Inhibition of Nuclear Factor (erythroid-derived 2)-like 2 promotes hepatic progenitor cell activation

Authors

Francesco Bellanti[1,¶,*], Giorgia di Bello[1,¶], Giuseppina Iannelli[1], Giuseppe Pannone[2], Maria Carmela Pedicillo[2], Luke Boulter[3], Wei-Yu Lu[4], Rosanna Tamborra[1], Rosanna Villani[1], Gianluigi Vendemiale[1], Stuart J Forbes[5], and Gaetano Serviddio[1]

Affiliations

1. Liver Unit – C.U.R.E. Centre for Liver Disease Research and Treatment, Department of Medical and Surgical Sciences, University of Foggia, Foggia, Italy
2. Anatomical Pathology Unit, Department of Clinical and Experimental Medicine, University of Foggia, Foggia, Italy
3. MRC Human Genetics Unit, Institute of Genetics and Molecular Medicine, University of Edinburgh, Edinburgh, United Kingdom
4. Centre for Liver and Gastrointestinal Research, Institute of Immunology and Immunotherapy, University of Birmingham, Birmingham, United Kingdom
5. MRC Centre for Regenerative Medicine, University of Edinburgh, Edinburgh, United Kingdom

***Correspondence:** Francesco Bellanti, Department of Medical and Surgical Sciences, University of Foggia, Viale Pinto, 1 – 71122 – Foggia, Italy   email: francesco.bellanti@unifg.it

Tel/Fax 0039 0881 732671

This Supplementary Information file includes:

Supplementary Fig.1. Effect of pro-oxidants treatment on hepatic progenitor cells (HPCs).

Supplementary Fig. 2. Effect of H_2O_2 and N-acetylcysteine (NAC) treatment on EpCAM expression and apoptosis in hepatic progenitor cells (HPCs).

Supplementary Fig. 3. Gene Set Enrichment Analysis (GSEA) results of c2 Gene Ontology (GO) and Kyoto Encyclopaedia of Genes and Genomes (KEGG) reference gene sets.

Supplementary Fig. 4. Evaluation of the knockdown efficiency of NRF2 following siRNA transfection. NRF2 mRNA expression levels in primary mouse HPCs.

Supplementary Fig. 5. NRF2 protein amount in primary hepatocytes and hepatic progenitor cells (mHPCs) isolated from lean mice and models of HPC activation.

Supplementary Fig. 6. NRF2 localization in the liver of injured mice.

Supplementary Fig. 7. Double immunohistochemistry of NRF2 and CK-19.

Supplementary Fig. 8. Digital image analysis virtual dual staining related to images shown in Supplementary Fig. 7.

Supplementary Fig. 9. Validation of the NRF2 activation model in the biliary tract.

Supplementary Fig. 10. Effect of ARE expression modulator 1 (AEM1) on Phosphoinositide-3 Kinase-Akt and Extracellular Signal Regulated Kinases (ERK) pathways, and on Nuclear Respiratory Factor 2 (NRF2) expression in HepaRG cells.

Supplementary Fig. 11. *In vivo* detection of transplanted HepaRG cells.

Supplementary Fig. 12. Immunoistochemical detection of transplanted HepaRG cells in mouse liver.

Supplementary Fig. 13. FACS gating strategy to isolate BECs/HPCs from mouse liver.

Supplementary Fig. 14. Uncropped images of Western Blots.

Supplementary Table 1. Differentially expressed NRF2 target genes.

Supplementary Table 2. RT-PCR primer sequences.

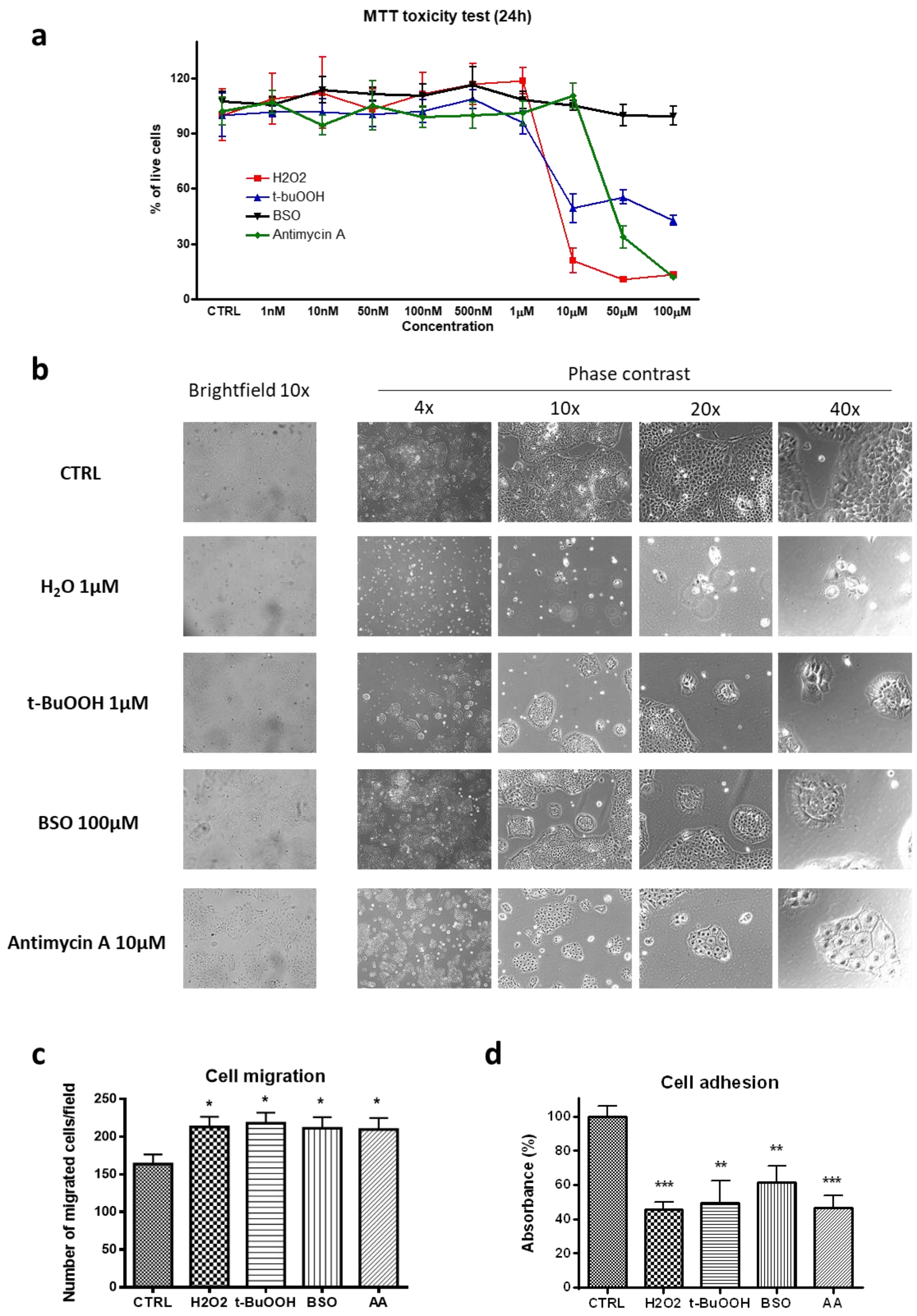

Supplementary Fig.1. Effect of pro-oxidants treatment on hepatic progenitor cells (HPCs). (a) HPCs were exposed to increasing concentrations of H_2O_2, tert-butyl hydroperoxide (t-BuOOH), buthionine sulfoximine (BSO), or antimycin A. Cell viability was measured by 3-(4,5-dimethylthiazol-2-yl)-2,5diphenyltetrazolium bromide (MTT) reduction after 24 hours. **(b)** Representative microscopic images of HPCs exposed to the maximal non-toxic amount of pro-oxidants every 24 hours for 5 days. **(c)** Migration assay in HPCs exposed to the maximal non-toxic amount of pro-oxidants every 24 hours for 5 days. **(d)** Adhesion assay in HPCs exposed to the maximal non-toxic amount of pro-oxidants every 24 hours for 5 days. Data in the graphs are represented as mean ± SD of three independent experiments. Statistical differences were assessed by one-way ANOVA and Tukey as post hoc test. * = $p < 0.05$ vs CTRL; ** = $p < 0.01$ vs CTRL; *** = $p < 0.001$ vs CTRL.

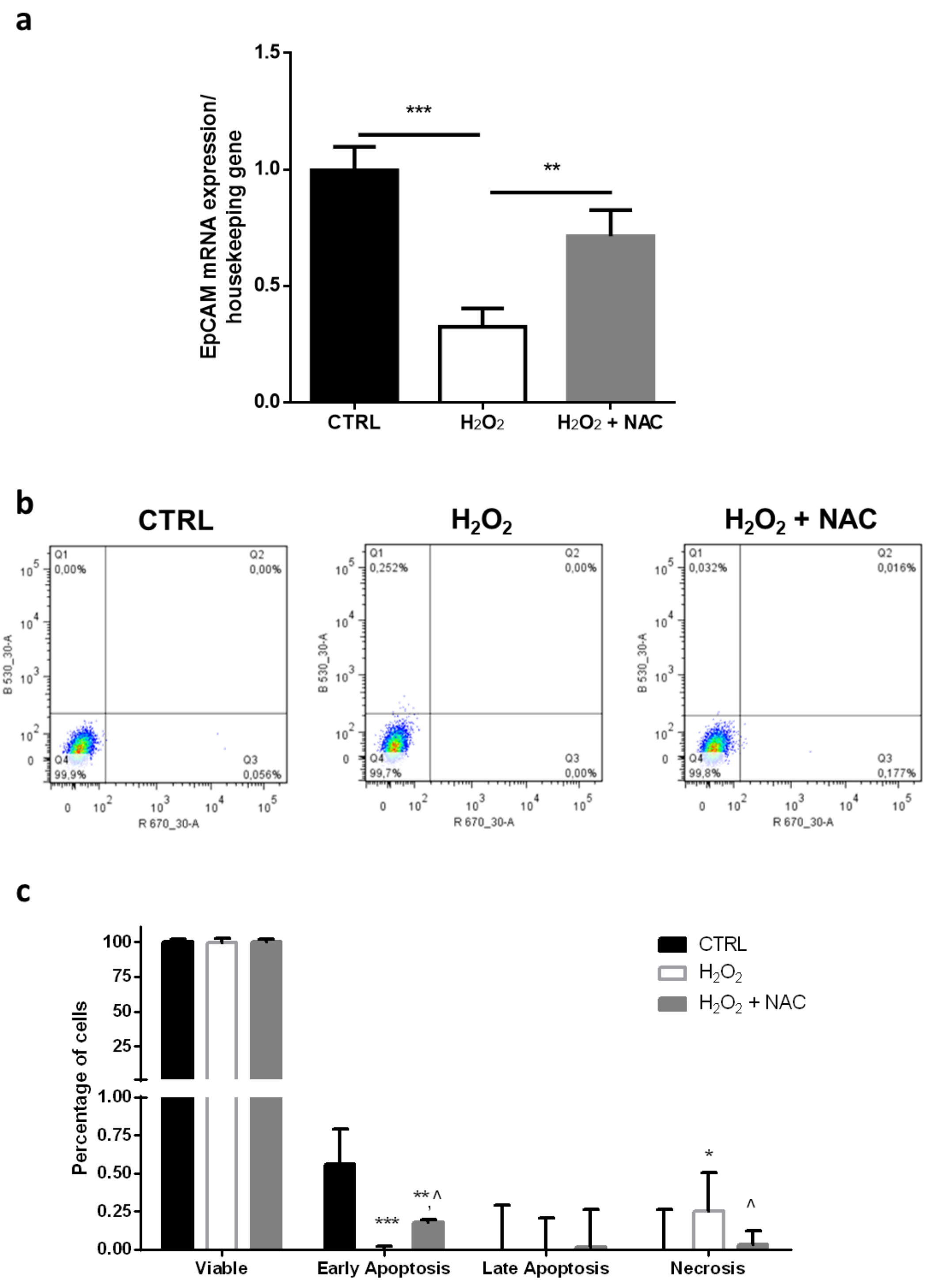

Supplementary Fig. 2. Effect of H_2O_2 and N-acetylcysteine (NAC) treatment on EpCAM expression and apoptosis in hepatic progenitor cells (HPCs). HPCs were exposed to 1 µM H_2O_2 or 1 µM H_2O_2 + 1 mM NAC for 24 hours. **(a)** EpCAM mRNA expression was analyzed by real time RT-PCR. **(b, c)** Apoptosis was assessed by flow cytometry after staining with annexin V (AV) and 7-Amino-Actinomycin D (7-AAD). Q1: AV negative/7-AAD positive cells (necrosis); Q2: AV/7-AAD positive cells (late apoptosis); Q3: AV positive/7-AAD negative cells (early apoptosis); Q4: AV/7-AAD negative cells (viable). Data in the graphs are represented as mean ± SD of three independent experiments. Statistical differences were assessed by one-way ANOVA and Tukey as post hoc test. * = $p < 0.05$ vs CTRL; ** = $p < 0.01$ vs CTRL; *** = $p < 0.001$ vs CTRL; ^ = $p < 0.05$ vs H_2O_2.

a

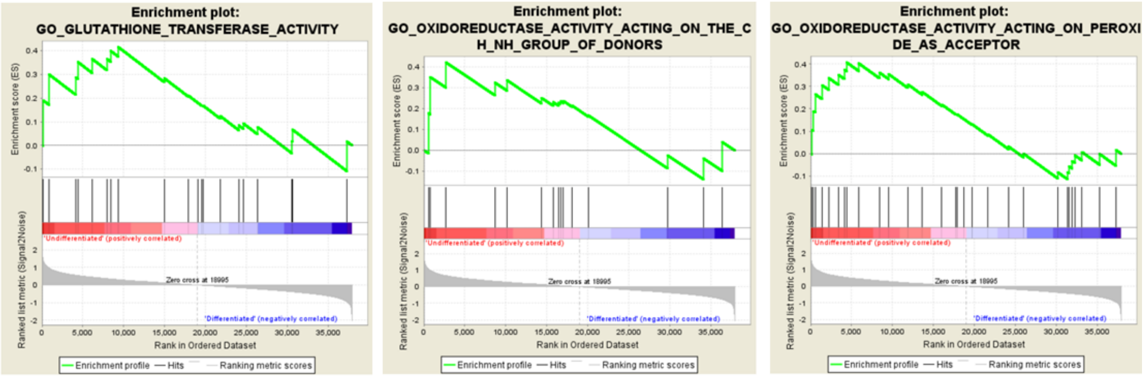

b

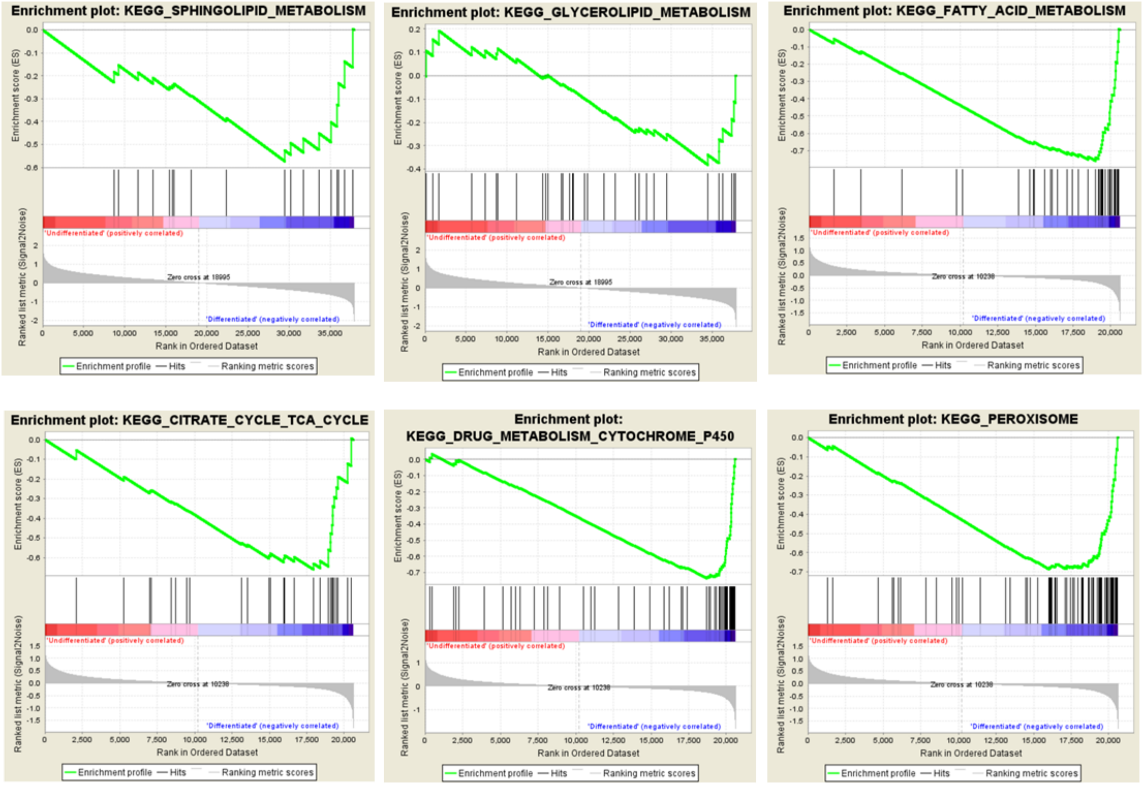

Supplementary Fig. 3. Gene Set Enrichment Analysis (GSEA) results of c2 Gene Ontology (GO) and Kyoto Encyclopaedia of Genes and Genomes (KEGG) reference gene sets. (a) GSEA plots for the up-regulated gene signatures in undifferentiated HPCs. **(b)** GSEA plots for down-regulated gene signatures in HPCs.

The top part of each plot shows the enrichment score that represents running-sum statistic calculated by "walking down" the ranked list of genes. The middle part shows the position of a member of a gene set in the ranked list of genes. The bottom part depicts the ranking metric that measures a gene's correlation with a biological function. NES, normalized enrichment score.

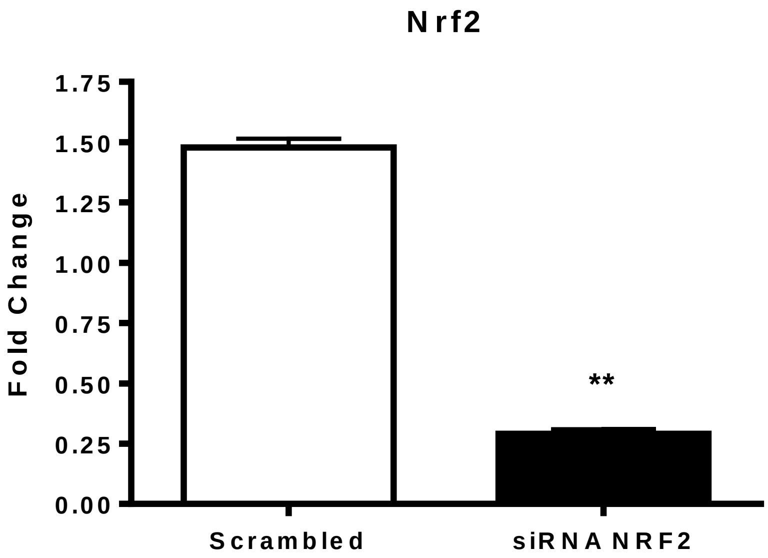

Supplementary Fig. 4. Evaluation of the knockdown efficiency of NRF2 following siRNA transfection. NRF2 mRNA expression levels in primary mouse HPCs. Values were normalized to endogenous actin mRNA. Data in the graphs are represented as mean ± SD of three independent experiments. Statistical differences were assessed by student's t-test. ** = $p < 0.01$ vs Scrambled.

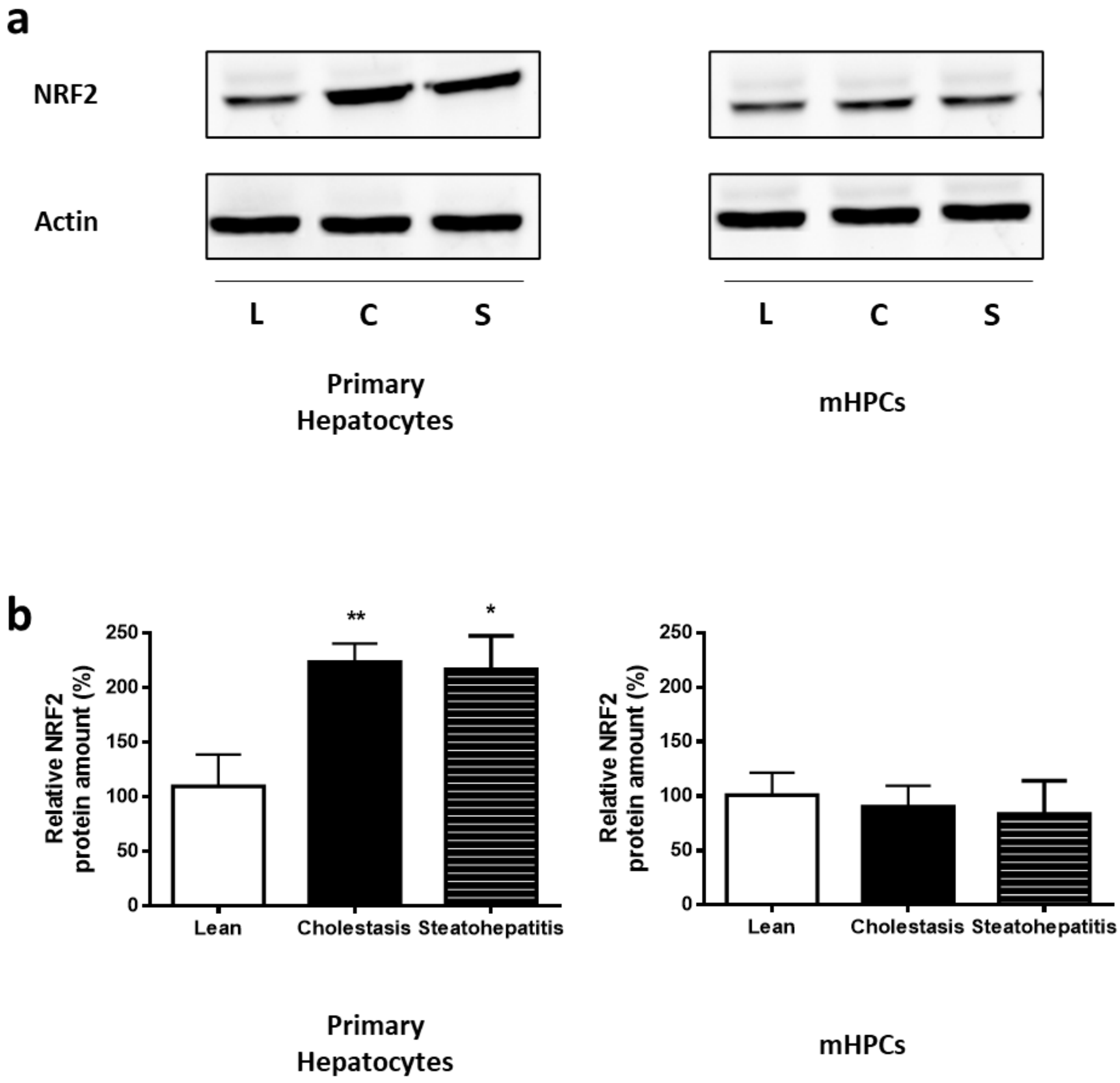

Supplementary Fig. 5. NRF2 protein amount in primary hepatocytes and hepatic progenitor cells (mHPCs) isolated from lean mice and models of HPC activation. (a) Representative Western blot and **(b)** relative densitometric analysis. All blots derive from the same experiment and were processed in parallel. Data in the graphs are represented as mean ± SD of three independent experiments. Statistical differences were assessed by one-way ANOVA followed by Tukey as post hoc test. * = $p < 0.05$ vs Lean, ** = $p < 0.001$ vs Lean. L = Lean; C = Cholestasis; S = Steatohepatitis.

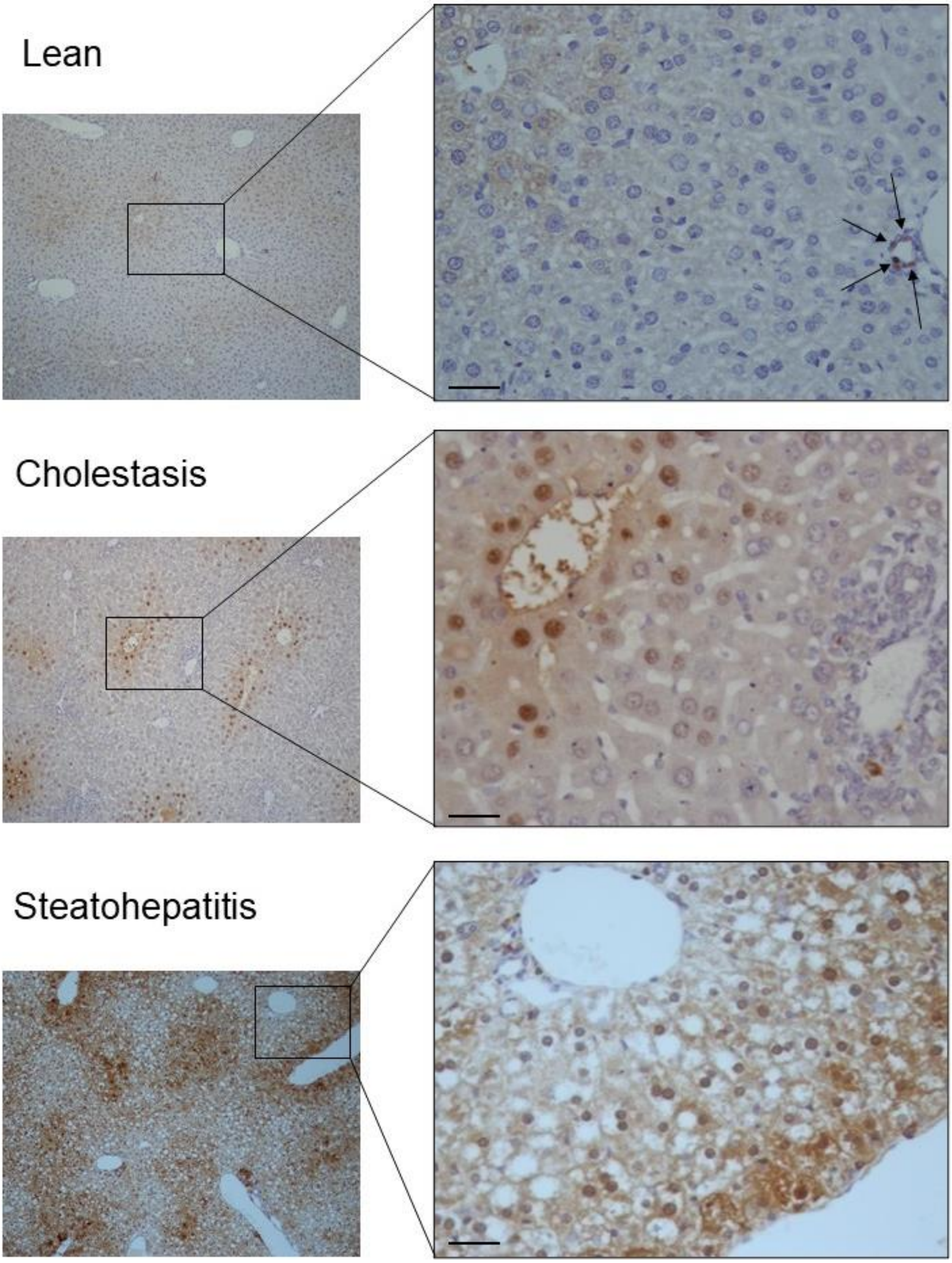

Supplementary Fig. 6. NRF2 localization in the liver of injured mice. Representative images showing immunohistochemical detection of NRF2 in the liver of mice fed a control chow (lean), 3,5-diethoxycarbonyl-1,4-dihydrocollidine diet (cholestasis), and methionine-choline deficient diet (steatohepatitis) (Left: magnification 50x; Right: magnification 200x).

Lean

Steatohepatitis

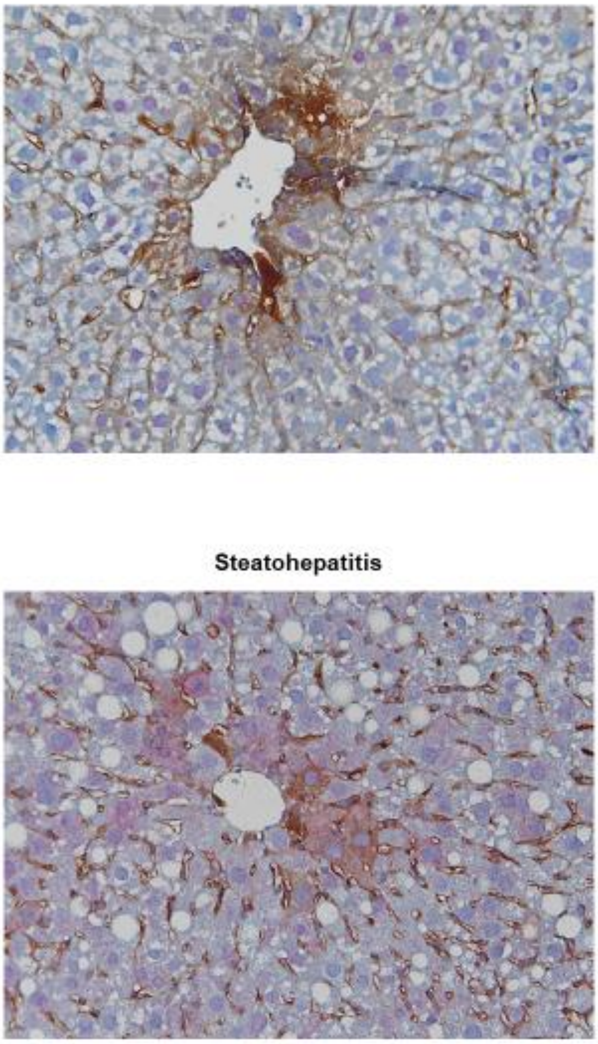

Supplementary Fig. 7. Double immunohistochemistry of NRF2 and CK-19. Representative images showing double immunohistochemical detection of NRF2 (red) and CK-19 (brown) in the liver of mice fed a control chow (lean) or methionine-choline deficient diet (steatohepatitis) (Magnification 200 x).

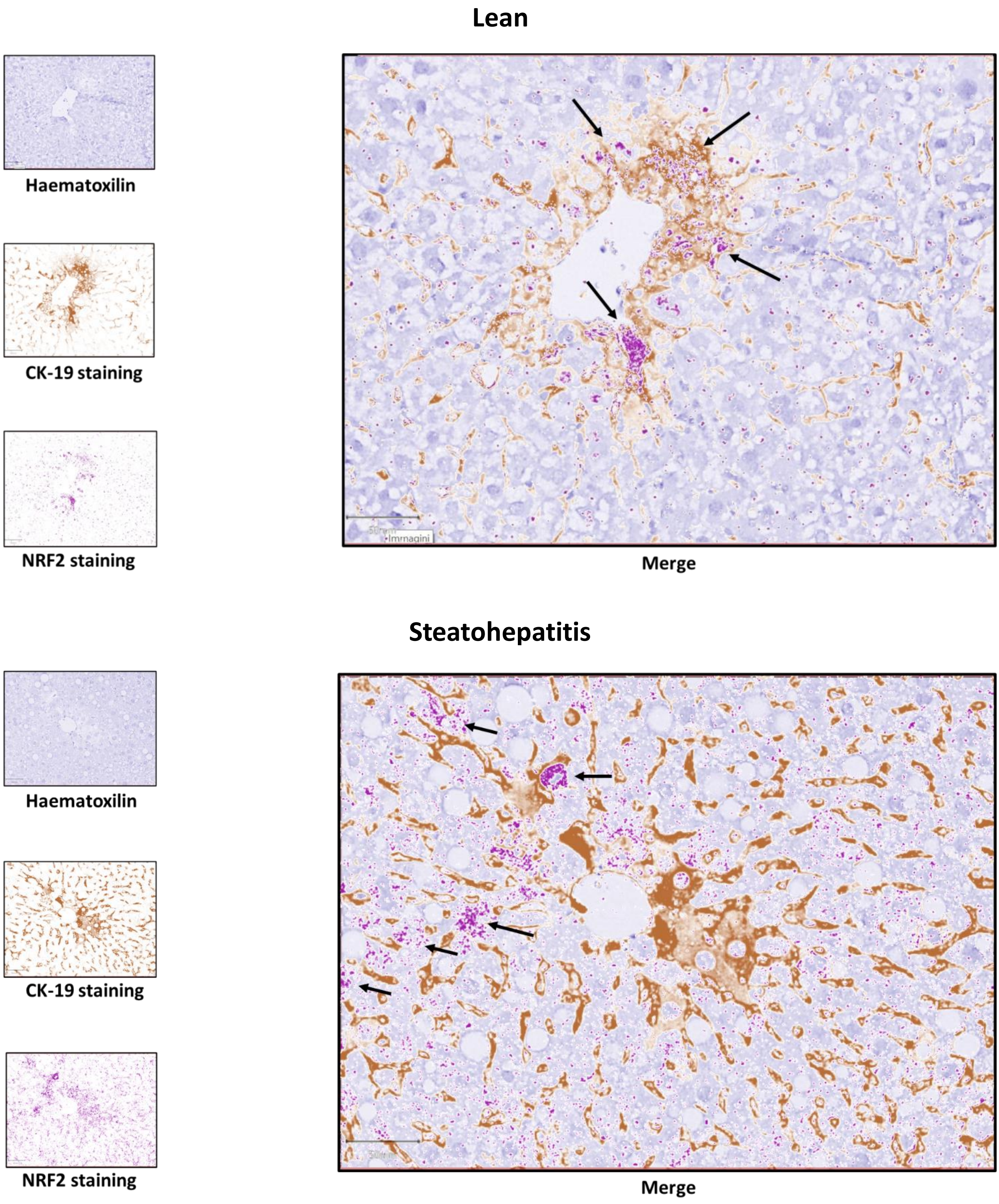

Supplementary Fig. 8. Digital image analysis virtual dual staining related to images shown in Supplementary Fig. 7. Corresponding cytokeratin-19 (CK-19, brown) and Nuclear Respiratory Factor 2 (NRF 2, violet) stains are virtually aligned. Arrows indicate NRF2 detection in ductular/HPCs (lean) or hepatocytes (steatohepatitis) (Magnification 200 x).

a

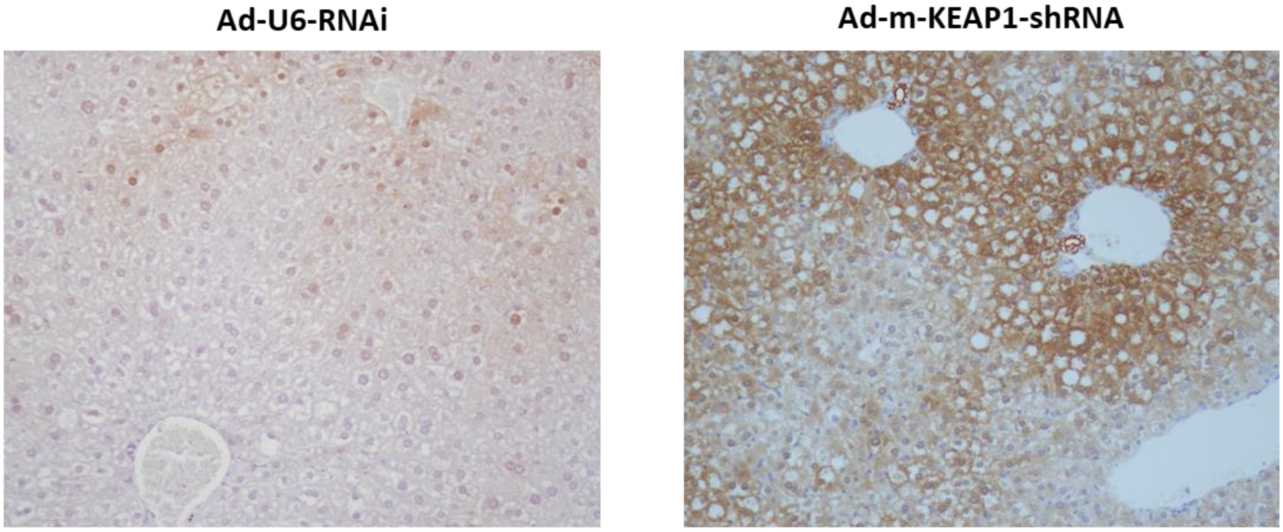

b

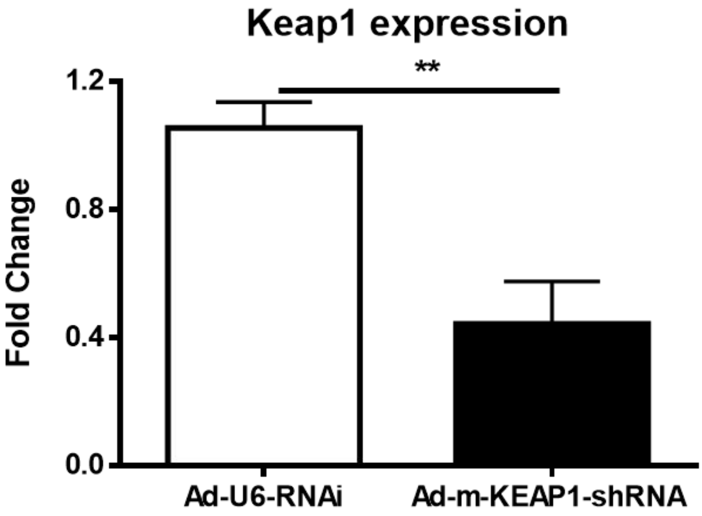

Supplementary Fig. 9. Validation of the NRF2 activation model in the biliary tract. (a) Representative images showing immunohistochemical detection of NRF2 in the liver of mice fed a methionine-choline deficient diet (steatohepatitis) and treated with Ad-m-KEAP1-shRNA, an adenovirus expressing shRNA for silencing of mouse Kelch-like ECH-associated protein 1 (Keap1, a repressor of NRF2), or Ad-U6-RNAi, an adenovirus expressing a scrambled shRNA as control (Magnification 200 x). **(b)** KEAP1 mRNA expression in the liver of mice from the same treatment groups. Data in the graphs are represented as mean ± SD of three independent experiments. Statistical differences were assessed by student's t-test. ** = $p < 0.01$ vs Ad-U6-RNAi.

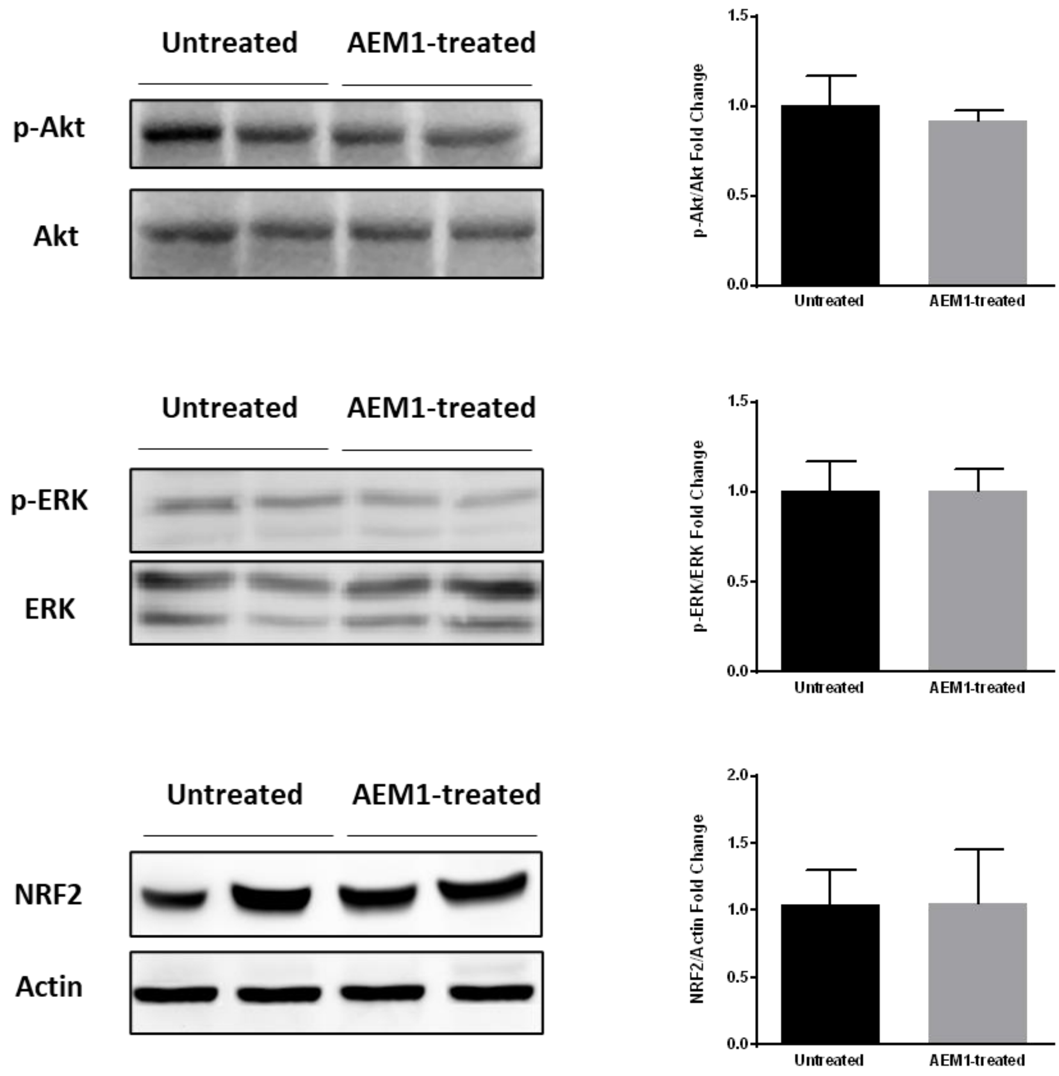

Supplementary Fig. 10. Effect of ARE expression modulator 1 (AEM1) on Phosphoinositide-3 Kinase-Akt and Extracellular Signal Regulated Kinases (ERK) pathways, and on Nuclear Respiratory Factor 2 (NRF2) expression in HepaRG cells. Representative western blots and relative densitometric analysis of phosphorylated and non-phosphorylated Akt and ERK, as well as NRF2, in HepaRG cells untreated or treated with AEM1. All blots derive from the same experiment and were processed in parallel. Data are expressed as mean ± SD of three separate experiments. Statistical differences were assessed using student’s t-test.

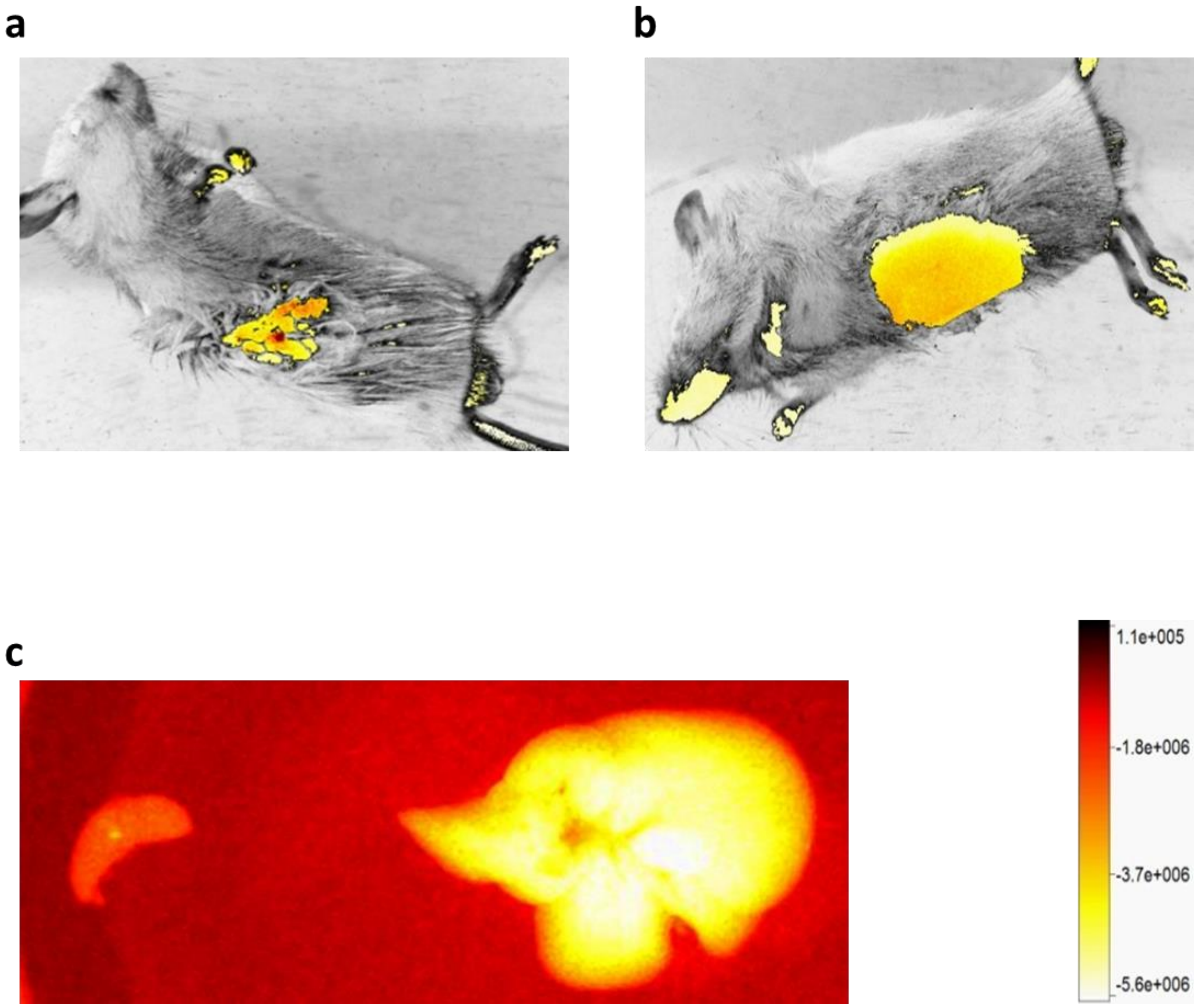

Supplementary Fig. 11. ***In vivo*** **detection of transplanted HepaRG cells.** Representative images showing **(a)** a SCID Beige mouse 15 minutes after the intrasplenic injection of 1.0 * 10^6 HepaRG cells labelled with 1, 1' – Dioctadecyl-3, 3, 3', 3'- Tetramethylindotricarbocyanine Iodide (DIR): the fluorescence is detectable on the upper left abdominal side; **(b)** the same SCID Beige mouse 24 hours after HepaRG transplantation: the fluorescence is mainly detectable on the upper right abdominal side; **(c)** spleen (left) and liver (right) excised from the same SCID Beige mouse 4 weeks after HepaRG transplantation.

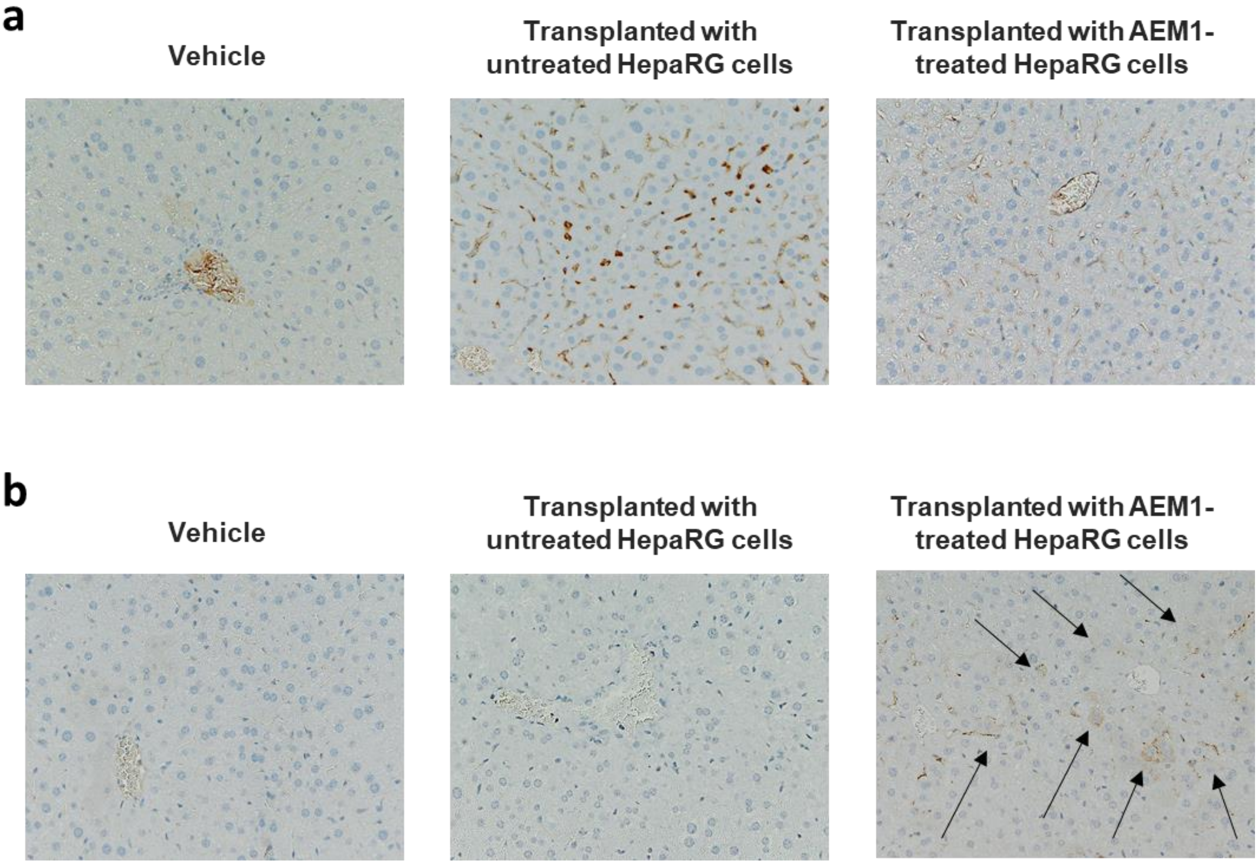

Supplementary Fig. 12. Immunoistochemical detection of transplanted HepaRG cells in mouse liver. (a) Representative images showing immunohistochemical detection of human Cytokeratin 7 in the liver of SCID Beige mice treated with Jo2 mAb once weekly for 4 consecutive weeks after transplantation (Magnification 200 x). **(b)** Representative images showing immunohistochemical detection of human albumin in the liver of SCID Beige mice treated with Jo2 mAb once weekly for 4 consecutive weeks after transplantation (Magnification 200 x).

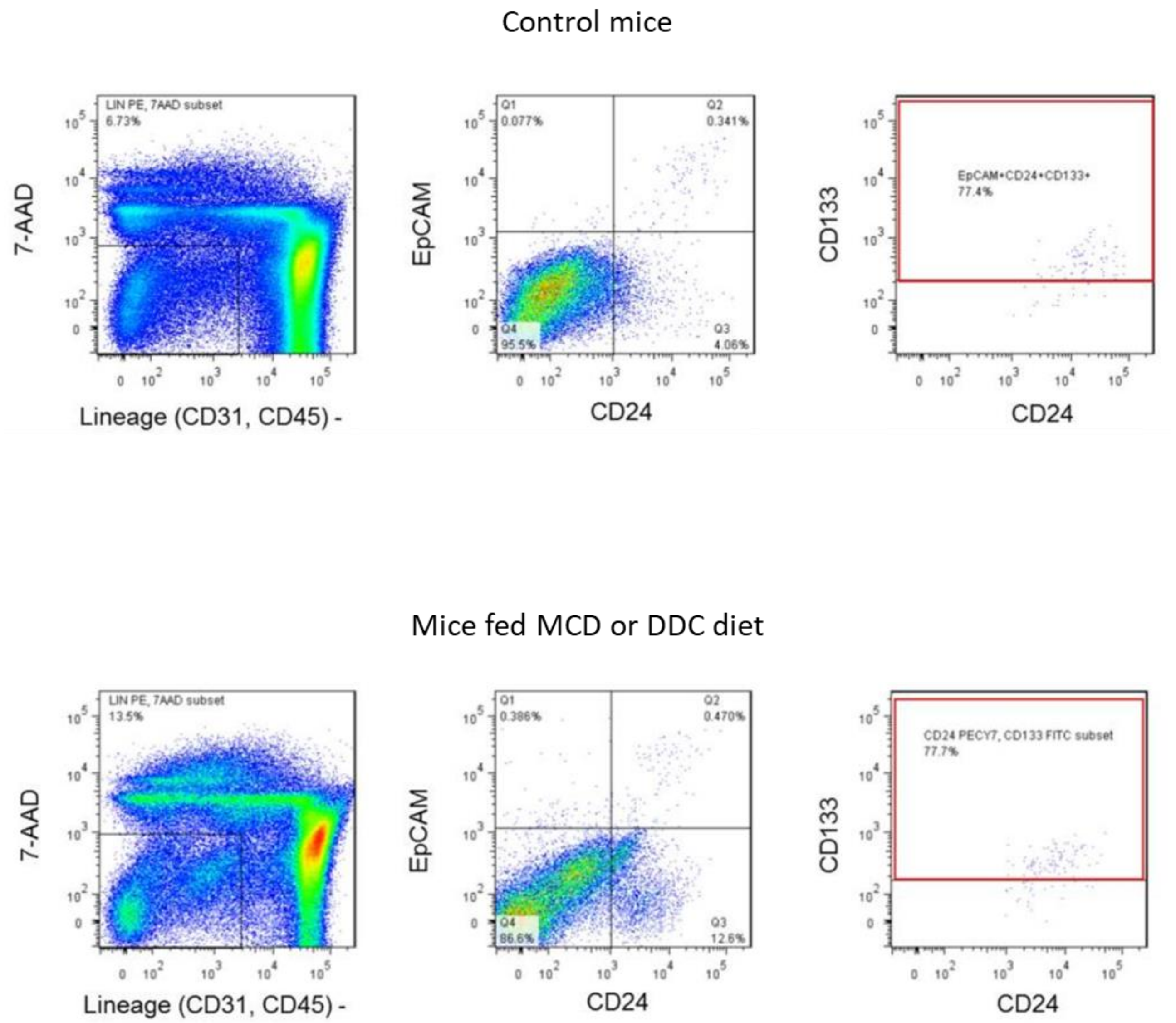

Supplementary Fig. 13. FACS gating strategy to isolate BECs/HPCs from mouse liver. FACS separation of BECs/HPCs was performed using EpCAM+/CD24+/CD133+/CD31-/CD45-/Ter119- sorting from lean mice and animals fed a methionine and choline deficient (MCD) or a 3,5-diethoxycarbonyl-1,4-dihydrocollidine (DDC) diet for up to 2 weeks.

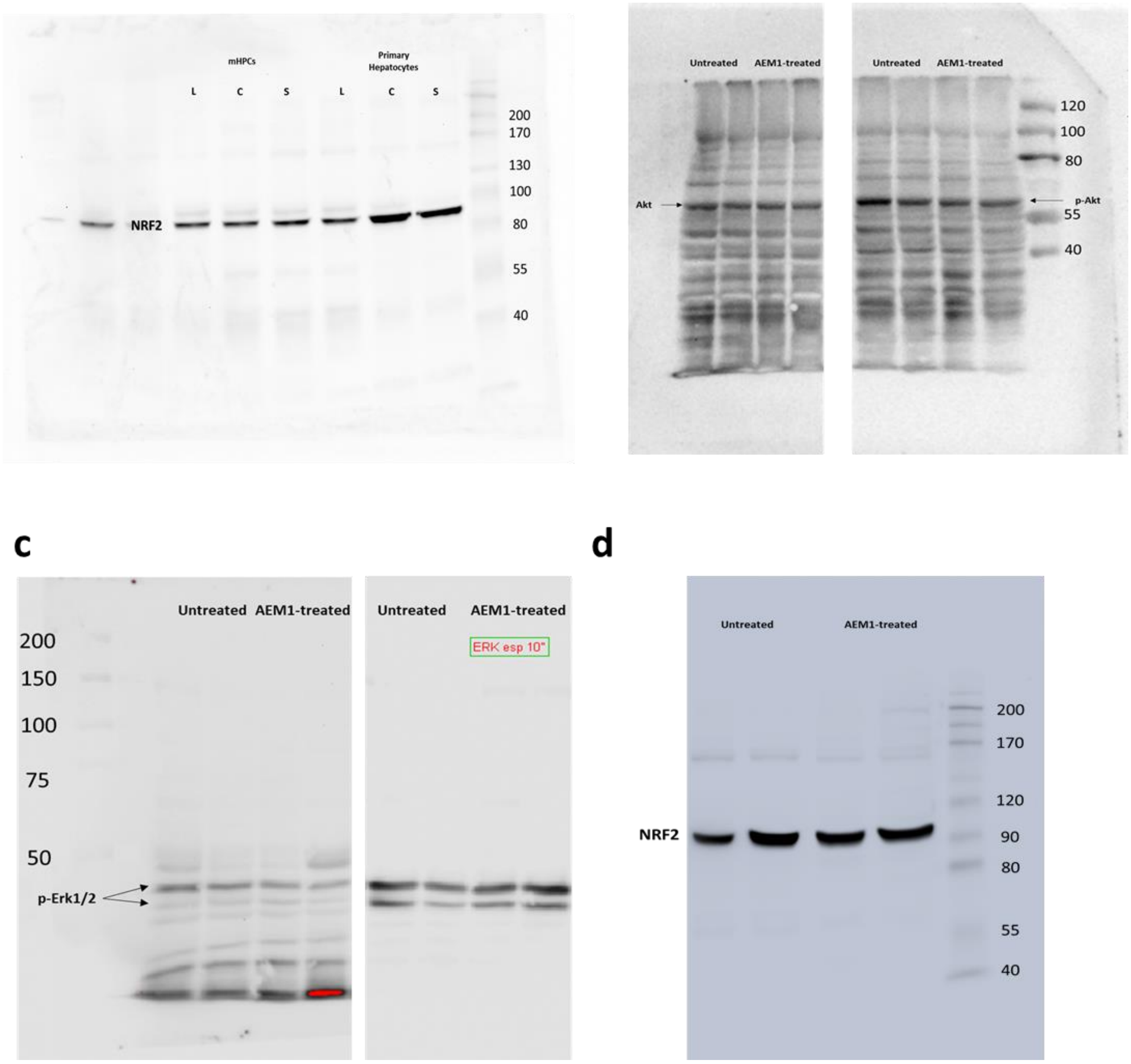

Supplementary Fig. 14. Uncropped images of Western Blots. Uncropped images from Western blots hat correspond to Supplementary Fig. 5 **(a)**, and Supplementary Fig. 9 **(b-d)**. All blots derive from the same experiment and were processed in parallel.

Supplementary Table 1. Differentially expressed NRF2 target genes. Up-regulated NRF2 target genes in common between GSE7038 and GSE28891 in undifferentiated hepatic progenitors with respect to differentiated cells.

Gene symbol	Gene title	Adj P Value	P Value	T	B	logFC
Mgst1	microsomal glutathione S-transferase 1	0.45	0.0135412	3.93	-2.43074	2.74
Abcc5	ATP-binding cassette, sub-family C (CFTR/MRP), member 5	0.517	0.0258566	3.27	-3.0555	2.24
Gsr	glutathione reductase	0.595	0.0441342	2.77	-3.58202	1.70
Nqo1	NAD(P)H dehydrogenase, quinone 1	0.431	0.011475	4.11	-2.27419	2.30
Hmox1	heme oxygenase 1	0.683	0.0783972	2.27	-4.14928	1.25

Supplementary Table 2. RT-PCR primer sequences.

Gene name	Specie	Forward/Reverse	Sequences
**ACTIN**	Mouse	FOR	5'-GGCGGGACTGTTACTGAGCTGCG-3'
		REV	5'-GCTGTCGCCTTCACCGTTCCA-3'
**HMOX1**	Mouse	FOR	5'-CCCTGCTGAGTAATCCTTTCCCGA-3'
		REV	5'-ATGTCCCGACTCCAGACTCCA-3'
**NQO1**	Mouse	FOR	5'-GTGGAAGTCGTCCCAAGAGA-3'
		REV	5'-TGTCTCCCCAGGACTCTCTCAG-3'
**KEAP1**	Mouse	FOR	5'-GGCTGTCCTCAATCGTCTCC-3'
		REV	5'-TCTGTTTCCACATCGTAGCG-3'
**ACTIN**	Human	FOR	5'-TGGACATCCGCAAAGACCTG-3'
		REV	5'-GCCGATCCACACGGAGTACTT-3'
**ALBUMIN**	Human	FOR	5'-CCTGTTGCCAAAGCTCGATG-3'
		REV	5'-GAAATCTCTGGCTCAGGCGA-3'
**CYP3A4**	Human	FOR	5'-CTTCATCCAATGGACTGCATAAAT-3'
		REV	5'-TCCCAAGTATAACACTCTACACAG-3'
**CEA**	Human	FOR	5'-GGTCTTCAACCCAATCAGTAAGAAC-3'
		REV	5'-ATGGCCCCAGGTGAGAGG-3'
**GGT-1**	Human	FOR	5'-TTTGGTGTGCTGCTGGATGAC-3'
		REV	5'-ACCTGAGCTTCCCCACCTATG-3'
**CK**	Human	FOR	5'-TCCGAACCAAGTTTGAGACG-3'
		REV	5'-GCCCCTCAGCGTACTGATTT-3'
**SOD1**	Human	FOR	5'-TGTGGGGAAGCATTAAAGG-3'
		REV	5'-CCGTGTTTTCTGGATAGAGG-3'
