# Supplementary material for: Inhibition of nuclear factor (erythroid-derived 2)-like 2 promotes hepatic progenitor cell activation and differentiation
Source: NPJ Regen Med. 2021 May 26;6:28. doi: 10.1038/s41536-021-00137-z (PMC8155039; doi:10.1038/s41536-021-00137-z)
Supplement: Supplementary file 2 — Reporting Summary [file 41536_2021_137_MOESM2_ESM.pdf]

nature research

Corresponding author(s): Francesco Bellanti

Last updated by author(s): Dec 4, 2020

Reporting Summary

Nature Research wishes to improve the reproducibility of the work that we publish. This form provides structure for consistency and transparency in reporting. For further information on Nature Research policies, see our Editorial Policies and the Editorial Policy Checklist.

Statistics

For all statistical analyses, confirm that the following items are present in the figure legend, table legend, main text, or Methods section.

n/a	Confirmed	
☐	☒	The exact sample size (*n*) for each experimental group/condition, given as a discrete number and unit of measurement
☐	☒	A statement on whether measurements were taken from distinct samples or whether the same sample was measured repeatedly
☐	☒	The statistical test(s) used AND whether they are one- or two-sided<br>*Only common tests should be described solely by name; describe more complex techniques in the Methods section.*
☒	☐	A description of all covariates tested
☒	☐	A description of any assumptions or corrections, such as tests of normality and adjustment for multiple comparisons
☐	☒	A full description of the statistical parameters including central tendency (e.g. means) or other basic estimates (e.g. regression coefficient) AND variation (e.g. standard deviation) or associated estimates of uncertainty (e.g. confidence intervals)
☐	☒	For null hypothesis testing, the test statistic (e.g. *F*, *t*, *r*) with confidence intervals, effect sizes, degrees of freedom and *P* value noted<br>*Give P values as exact values whenever suitable.*
☒	☐	For Bayesian analysis, information on the choice of priors and Markov chain Monte Carlo settings
☒	☐	For hierarchical and complex designs, identification of the appropriate level for tests and full reporting of outcomes
☒	☐	Estimates of effect sizes (e.g. Cohen's *d*, Pearson's *r*), indicating how they were calculated

Our web collection on statistics for biologists contains articles on many of the points above.

Software and code

Policy information about availability of computer code

Data collection	GraphPad Prism 6.0 software (San Diego, CA)
Data analysis	GraphPad Prism 6.0 software (San Diego, CA)

For manuscripts utilizing custom algorithms or software that are central to the research but not yet described in published literature, software must be made available to editors and reviewers. We strongly encourage code deposition in a community repository (e.g. GitHub). See the Nature Research guidelines for submitting code & software for further information.

Data

Policy information about availability of data

All manuscripts must include a data availability statement. This statement should provide the following information, where applicable:

- Accession codes, unique identifiers, or web links for publicly available datasets
- A list of figures that have associated raw data
- A description of any restrictions on data availability

All relevant data supporting the findings of this study are available within the paper, its supplementary information and from the corresponding authors upon reasonable request.

Field-specific reporting

Please select the one below that is the best fit for your research. If you are not sure, read the appropriate sections before making your selection.

☒ Life sciences ☐ Behavioural & social sciences ☐ Ecological, evolutionary & environmental sciences

For a reference copy of the document with all sections, see nature.com/documents/nr-reporting-summary-flat.pdf

Life sciences study design

All studies must disclose on these points even when the disclosure is negative.

Sample size	No sample size calculations were performed. The sample size (n) of each experiment is provided in the corresponding figure captions in the main manuscript and supplementary information files. Sample sizes were chosen to support meaningful conclusions.
Data exclusions	No data was excluded from the analysis.
Replication	All in vitro experiments were replicated successfully 3 times.
Randomization	Randomization was not necessary for this study.
Blinding	Blinding was not relevant to this study.

Reporting for specific materials, systems and methods

We require information from authors about some types of materials, experimental systems and methods used in many studies. Here, indicate whether each material, system or method listed is relevant to your study. If you are not sure if a list item applies to your research, read the appropriate section before selecting a response.

Materials & experimental systems

n/a	Involved in the study	
☐	☒	Antibodies
☐	☒	Eukaryotic cell lines
☒	☐	Palaeontology and archaeology
☐	☒	Animals and other organisms
☒	☐	Human research participants
☒	☐	Clinical data
☒	☐	Dual use research of concern

Methods

n/a	Involved in the study	
☒	☐	ChIP-seq
☐	☒	Flow cytometry
☒	☐	MRI-based neuroimaging

Antibodies

Antibodies used	Anti-NRF2 primary antibody (Abcam, ab31163); AlexaFluor 488-conjugated secondary antibody (Abcam, ab150073); PE-labelled antibodies against CD34 (1:50, 130-081-002), CD184 (CXCR4; 1:10, 130-098-354), CD49a (1:50, 130-101-397), CD49f (1:50, 130-097-246), CD326 (EpCAM; 1:50, 130-091-253), rat IgG1κ isotype control (1:10, 130-102-645), and FITC-labelled CK (1:11, 130-080-101) by Miltenyi Biotec; primary mouse polyclonal anti-human CK19 (Ventana, 760-4281); primary mouse monoclonal anti-human CK7 (OV-TL 12/30, Cell Marque, CMC30729050); primary mouse monoclonal anti-human Glypcan3 (Ventana, 790-4564); primary mouse monoclonal anti-human albumin (MyBioSource, MBS766511); primary rabbit anti-mouse hydroxynonenal (HNE) (1:200; Abcam, ab46545); primary rabbit anti-mouse NRF2 (1:150; Abcam, ab31163); rabbit anti-NRF2 primary antibody (1:1000; Abcam, ab137550); rabbit anti-phospho-Akt (Ser473) (Abcam ab81283); rabbit anti-Akt (Abcam, ab126811); rabbit anti-phospho-ERK1/2 (Thr202 + Tyr204) (Abcam, ab214362), rabbit anti-ERK1/2 (Abcam, ab196883), rabbit anti-β-actin (Abcam, ab8227); goat HRP-conjugated anti-rabbit secondary antibody (1:2000; Abcam, ab205718).
Validation	All the antibodies were validated for species and application by the manufacturers.

Eukaryotic cell lines

Policy information about cell lines

Cell line source(s)	Primary ductular/HPCs were extracted by lean C57Bl6 mice and rodent models of ductular/HPC activation.<br>The human cell line HepaRG was purchased by Merck Millipore (MMHPR116).
Authentication	FACS separation of ductular/HPCs was performed using EpCAM+/CD24+/CD133+/CD31-/CD45-/Ter119- sorting. FACS characteristics of ductular/HPCs from uninjured and injured liver were described previously (Wei YL et al., Nat Cell Biol. 2015

	Aug; 17(8): 971–983).<br>HepaRG cells were authenticated by the manufacturer.
Mycoplasma contamination	All cell lines tested negative for mycoplasma contamination.
Commonly misidentified lines<br>(See ICLAC register)	No misidentified cell lines were used.

Animals and other organisms

Policy information about studies involving animals; ARRIVE guidelines recommended for reporting animal research

Laboratory animals	6-8 months old male C57BL6/J mice.
Wild animals	The study did not involve wild animals.
Field-collected samples	The study did not involve samples collected on the field.
Ethics oversight	University of Foggia ethics committee

Note that full information on the approval of the study protocol must also be provided in the manuscript.

Flow Cytometry

Plots

Confirm that:

☒ The axis labels state the marker and fluorochrome used (e.g. CD4-FITC).

☒ The axis scales are clearly visible. Include numbers along axes only for bottom left plot of group (a 'group' is an analysis of identical markers).

☒ All plots are contour plots with outliers or pseudocolor plots.

☒ A numerical value for number of cells or percentage (with statistics) is provided.

Methodology

Sample preparation	1 million cells/sample were resuspended in PBS and left 10 min at 4°C in the dark; then, cells were centrifuged at 300 g for 10 min, and washed twice with PBS. Finally, samples were resuspended in PBS and analysed. Cells labelled with anti-CK were permeabilised with PBS + 0.1% X-100 Triton before staining.
Instrument	FlowSight Cytometer (Amnis, Merck Millipore)
Software	IDEAS software
Cell population abundance	No post-sort fraction.
Gating strategy	Using the FSC/SSC gating, debris was removed by gating on the main cell population. Positivity threshold for each cell line was defined on the basis negative sample. Identical positivity threshold was applied to all samples within cell line.

☒ Tick this box to confirm that a figure exemplifying the gating strategy is provided in the Supplementary Information.
